# FMT combined with atezolizumab-bevacizumab in hepatocellular carcinoma refractory to immunotherapy: an open label, single-arm, phase II pilot study

**DOI:** 10.1038/s41467-026-78219-z

**Published:** 2026-10-07

**Authors:** Katharina Pomej, Adrian Frick, Bernhard Scheiner, Maximilian Baumgartner, Klaus G. Schmetterer, Carlos S. Reyna-Blanco, Lorenz Balcar, Larissa Pajancic, Abelina Kreuter, Joana Séneca, Bela Hausmann, Thomas Köcher, Melanie Prinzensteiner, Katharina Lampichler, Nataliya Rohr-Udilova, Adrija Chakrabarty, Anton Klotz, Annika C. Lampl, Kerstin Zinober, Katharina Regnat, Michael Trauner, David Berry, Dietmar Tamandl, Thomas Vogl, Christoph Gasche, Matthias Pinter

**Affiliations:** 1 Division of Gastroenterology and Hepatology, Department of Medicine III, Medical University of Vienna, Vienna, Austria; 2 Vienna Liver Cancer Study Group, Division of Gastroenterology and Hepatology, Department of Medicine III, Medical University of Vienna, Vienna, Austria; 3 CeMM Research Center for Molecular Medicine of the Austrian Academy of Sciences, Vienna, Austria; 4 Department of Laboratory Medicine, Medical University of Vienna, Vienna, Austria; 5 Center for Cancer Research, Medical University of Vienna, Vienna, Austria; 6 Center for Microbiology and Environmental Systems Science, Department of Microbiology and Ecosystem Science, Division of Microbial Ecology, University of Vienna, Vienna, Austria; 7 Joint Microbiome Facility of the Medical University of Vienna and the University of Vienna, Vienna, Austria; 8 Division of Clinical Microbiology, Department of Laboratory Medicine, Medical University of Vienna, Vienna, Austria; 9 Metabolomics Service and Research Facility, Vienna Biocenter Core Facilities, Vienna, Austria; 10 Department of Biomedical Imaging and Image-guided Therapy, Medical University of Vienna, Vienna, Austria

**Keywords:** Hepatocellular carcinoma, Hepatocytes

## Abstract

Fecal microbiota transplantation (FMT) may help to overcome resistance to immunotherapy. In this single-center, single-arm phase II hypothesis-generating pilot study (FAB-HCC), we included 12 patients with advanced hepatocellular carcinoma (HCC) who progressed on atezolizumab plus bevacizumab. Patients received a single FMT via colonoscopy from a donor with HCC who responded to anti-PD-1 treatment or a healthy donor, combined with atezolizumab plus bevacizumab. The primary endpoint was safety as measured by incidence and severity of treatment-related adverse events (TRAEs). Secondary endpoints included radiological response and survival. Grade 3-4 TRAEs included arterial hypertension (*n* = 5) and proteinuria (*n* = 3), both related to bevacizumab, and diarrhea (*n* = 1), related to FMT. No serious TRAEs were observed. Objective response rate was 33% (*n* = 4/12) and median progression-free survival was 6.9 (95%CI, 3.0-10.8) months. Following FMT, recipient microbiome composition appeared more similar to that of the donor, and healthy donor FMT seemed to be associated with higher engraftment. FMT appeared to be associated with enrichment of response-associated and loss of deleterious gut bacteria. Circulating PD-1^+^CD8^+^ T cells decreased and CD32b^+^CD14^high^CD16^low^ monocytes increased following FMT plus immunotherapy. Phage-display immunoprecipitation sequencing revealed that antibody repertoire stability, rather than global diversity or composition, changed over time. Taken together, FMT combined with atezolizumab plus bevacizumab was safe and associated with a high response rate in patients with atezolizumab plus bevacizumab-refractory HCC. Given the limited sample size, these findings need cautious interpretation and confirmation in larger, adequately powered trials. ClinicalTrials.gov identifier: NCT05750030.

## Abstract (web summary)

In this open label, single-arm, phase II pilot study, the authors report that fecal microbiota transplantation combined with atezolizumab/bevacizumab is safe and associates with a high response rate in patients with atezolizumab/bevacizumab-refractory hepatocellular carcinoma.

## Introduction

The systemic treatment landscape of hepatocellular carcinoma (HCC), the most common primary liver cancer^1^, has changed remarkably over the last 5 years. While tyrosine kinase inhibitors were used to treat advanced HCC for more than a decade, immune checkpoint inhibitor (ICI)-based combinations represent the current standard of care in the first-line setting^2^. These immune-based combination therapies can induce radiological responses in around one-fifth to one-third of patients, with a median duration of response of up to 30 months^3–5^, and these responses translate into unprecedented survival rates^6–8^.

Atezolizumab plus bevacizumab was the first ICI-based combination that improved overall survival versus sorafenib—the previous standard of care—in a phase III randomized controlled trial^9^, and subsequently became the new reference standard in systemic front-line treatment of HCC^2^. Despite an encouraging objective response rate of around 30%, almost 20% of the patients have a primary resistance to atezolizumab plus bevacizumab, and the vast majority develop progressive disease at some point during treatment^4^.

The gut microbiome has been linked to response and resistance to cancer immunotherapy^10,11^. Hence, modulation of the gut microbiota through interventions such as fecal microbiota transplantation (FMT) may help to improve response to immunotherapy or even overcome resistance^12,13^.

Indeed, in two studies including patients with melanoma refractory to prior immunotherapy, reinduction of immunotherapy after FMT from anti-programmed cell death protein 1 (PD-1)-responding donors led to a response in 3 out of 10 and 3 out of 15 patients, respectively^14,15^. However, in a clinical trial including 13 patients with nivolumab-refractory advanced gastrointestinal cancers who received responder-derived FMT in combination with nivolumab, only one partial response was recorded^16^.

Gut dysbiosis is common in patients with chronic liver diseases and liver cancer^17^, and associated with immunosuppression and impaired antitumor immune surveillance in HCC^18,19^, making liver cancer an attractive target for gut microbiota modulation. In this single-center, open-label, single-arm, phase II pilot study, we evaluate the safety and efficacy of FMT in combination with atezolizumab plus bevacizumab in patients with HCC who progressed during prior treatment with atezolizumab plus bevacizumab. We show that this combination is safe and appears to be effective in overcoming resistance to atezolizumab plus bevacizumab in a subset of patients. We also describe longitudinal changes in gut microbiome composition, bile acid metabolites, peripheral blood mononuclear cells (PBMC), as well as IgG antibody epitope repertoires.

## Results

### Trial design and patient enrollment

**Fig. 1:**
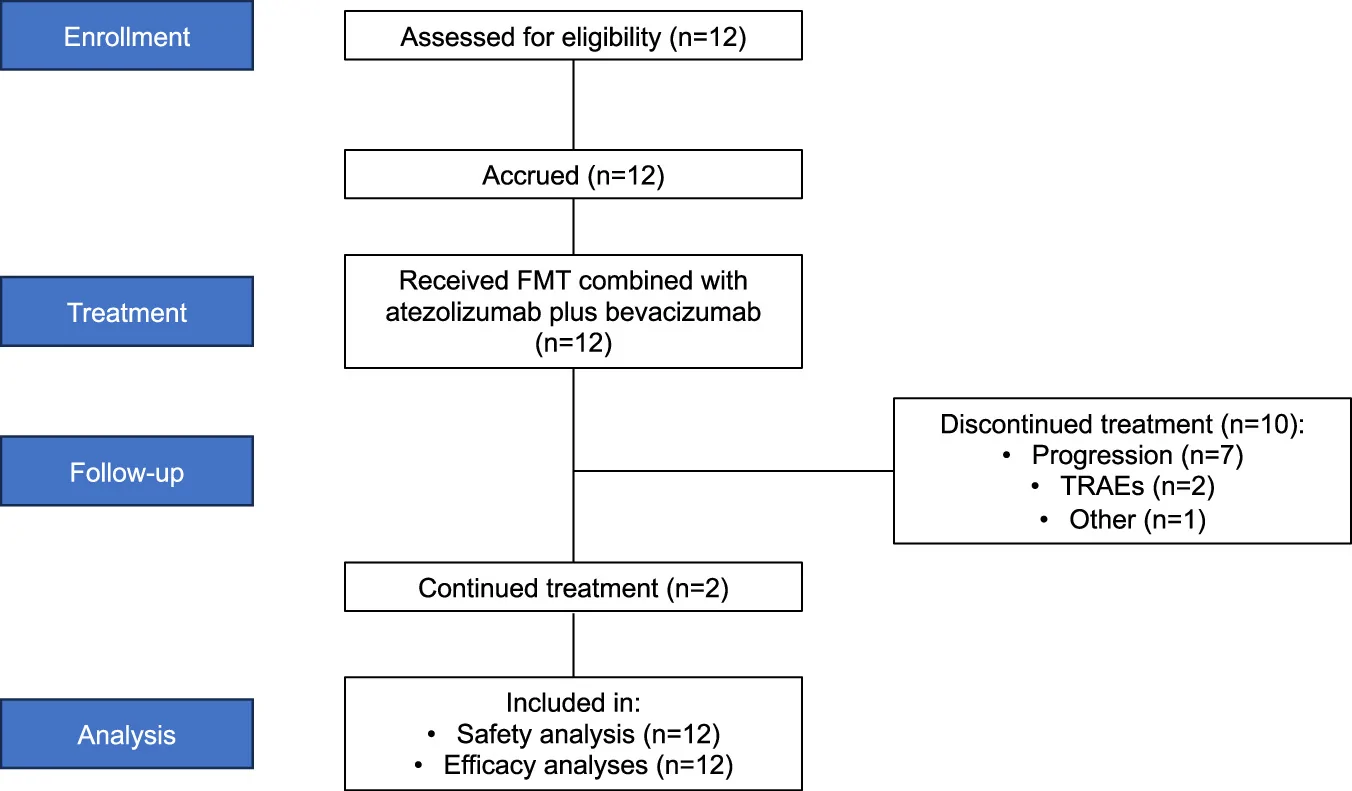
Study flow diagram. The diagram shows the number of patients screened, enrolled, treated, and analyzed. FMT fecal microbiota transplantation, TRAEs treatment-related adverse events.

Twelve patients were included in this single-center, open-label, single-arm phase II pilot study (NCT05750030) between August 2023 and November 2024. Patients with HCC who progressed on prior atezolizumab plus bevacizumab received a single FMT via colonoscopy from a donor with HCC who responded to anti-PD-1 treatment or a healthy donor, combined with atezolizumab plus bevacizumab. Patients were enrolled and treated at the Division of Gastroenterology & Hepatology, Medical University of Vienna. Patients were either screened and recruited at our outpatient clinic or referred from other hospitals in Vienna and the surrounding areas. The primary endpoint was safety as measured by incidence and severity of treatment-related adverse events (TRAEs). Secondary endpoints included best radiological response, objective response rate (ORR), disease control rate (DCR), progression-free survival (PFS), and overall survival (OS). A study flow diagram is provided in Fig. 1. Baseline characteristics are shown in Table 1 and individual patient characteristics are provided in Supplementary Table 1. Median time from last dose of prior atezolizumab to trial FMT was 1.2 (95% CI, 0.7–1.6) months. The first 6 patients received FMT from a donor with HCC who responded to anti-PD-1 treatment, and the other 6 individuals received FMT from a healthy donor. Clinical characteristics of FMT donors are shown in Supplementary Table 2. FMT per protocol (i.e., a total of 100 mL of fecal implant suspension administered in equal amounts of 20 mL to the terminal ileum, cecum, right colon, transverse colon, descending colon) was successfully achieved in 9 patients (75%), while the terminal ileum could not be intubated in 2 patients, and the right colon flexure could not be passed in 1 patient due to technical difficulties related to a challenging examination. In the latter 3 patients, all 100 mL of fecal implant suspension were evenly distributed over the sections reached during colonoscopy. All patients received at least one dose of atezolizumab plus bevacizumab.

**Table 1:** Baseline characteristics in all patients (*n* = 12)

Age, years	
Mean ± SD	68.4 ± 6.7
*Sex*	
Male	10 (83.3%)
Female	2 (16.7%)
*Etiology*	
HCV	6 (50%)
MASLD	3 (25%)
MetALD	1 (8.3%)
Unknown	2 (16.7%)
*ECOG performance status*	
0	12 (100%)
*Child-Pugh class*	
A	11 (91.7%)
B	1 (8.3%)
*BCLC stage*	
B	2 (16.7%)
C	10 (83.3%)
*Macrovascular invasion*	
Yes	4 (33.3%)
No	8 (66.7%)
*Extrahepatic metastasis*	
Yes	8 (66.7%)
No	4 (33.3%)
*Alpha-fetoprotein (ng/mL)*	
<400	8 (66.7%)
≥400	4 (33.3%)
*Any prior treatment before first-line atezolizumab plus bevacizumab*	
Yes	10 (83.3%)
No	2 (16.7%)
*Any treatment between the start of prior atezolizumab plus bevacizumab and the study inclusion*	
Yes	5 (41.7%)
No	7 (58.3%)
*Time from last dose of prior atezolizumab to trial FMT, months*	
Median (95%CI)	1.2 (0.7–1.6)

Data are *n* (%).

*BCLC* Barcelona Clinic Liver Cancer, *ECOG* Eastern Cooperative Oncology Group, *FMT* fecal microbiota transplantation, *HCV* hepatitis C virus, *MASLD* metabolic dysfunction-associated steatotic liver disease, *MetALD* metabolic dysfunction and alcohol-related liver disease, *SD* standard deviation.

At the time of the clinical cutoff date (March 17, 2025), median estimated follow-up was 11.4 (95% CI, 7.6–15.3) months with two patients still on treatment. Among the 10 participants who permanently discontinued treatment, 7 did so due to radiological progression, 2 due to TRAEs, and 1 patient discontinued due to worsening of general status. Nine participants received at least one subsequent treatment, and 4 patients died by the clinical cut-off date. A patient-level visualization of the disease course is provided in Supplementary Fig. 1.

### Safety

The primary endpoint of this study was safety as measured by incidence and severity of treatment-related adverse events (TRAEs). All patients received FMT and at least one dose of trial treatment and were included in the safety analyses. Median duration of treatment was 4.1 (95% CI, 0.0–9.4) months for atezolizumab and 4.1 (95% CI, 2.2–6.1) months for bevacizumab. There were no peri-interventional complications related to colonoscopy. All 12 patients (100%) experienced at least one TRAE, and 5 individuals (41.7%) had grade 3–4 TRAEs. Most adverse events related to atezolizumab plus bevacizumab included arterial hypertension (*n* = 10; 83.3%) and proteinuria (*n* = 9; 75%) (Table 2). Only one patient required systemic corticosteroids to treat an immune-related adverse event (i.e., nephritis). Adverse events related to FMT (including antibiotic pretreatment) included bloating, constipation, and diarrhea, and were all low-grade, except for diarrhea grade 3 in one patient (Table 2). No transmission of any infectious organism from FMT was observed. Dose interruption of atezolizumab and bevacizumab due to treatment-related adverse events was performed in 3 and 5 subjects, respectively. Permanent discontinuation of atezolizumab and bevacizumab due to TRAEs was recorded in 2 and 4 patients, respectively. No serious TRAEs or treatment-related deaths were observed. No previously unreported immune-related or FMT-related adverse events were documented.

**Table 2:** Treatment-related adverse events in all treated patients (*n* = 12)

	Any grade	Grade 3–4
Any TRAE	12 (100%)	5 (41.7%)
Any serious TRAE	0	0
**FMT-related adverse events**^**a**^		
Bloating	2 (16.7%)	0
Constipation	2 (16.7%)	0
Diarrhea	2 (16.7%)	1 (8.3%)
**Atezolizumab-related adverse events**		
Mucositis	2 (16.7%)	0
Pruritus	1 (8.3%)	0
Hypothyroidism	1 (8.3%)	0
Hyperthyroidism	1 (8.3%)	0
Amylase or lipase increases	1 (8.3%)	0
Hepatitis	1 (8.3%)	0
Nephritis	1 (8.3%)	0
Arthritis	1 (8.3%)	0
**Bevacizumab-related adverse events**		
Arterial hypertension	10 (83.3)	5 (41.7%)
Proteinuria	9 (75%)	3 (25%)

Data are *n* (%).

*FMT* fecal microbiota transplantation, *TRAE* treatment-related adverse event.

^a^Including antibiotic pretreatment.

### Clinical efficacy

**Fig. 2:**
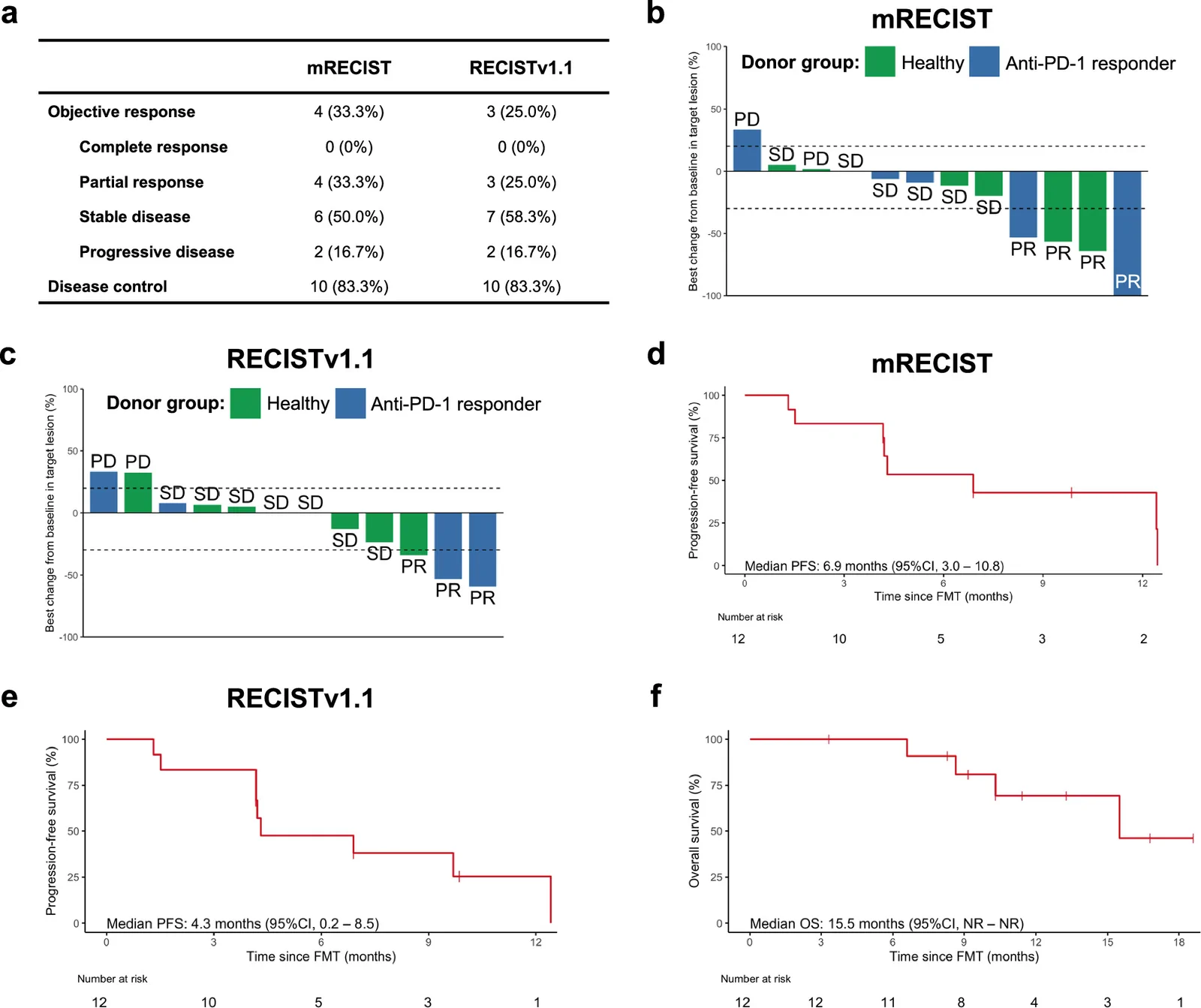
Main efficacy outcomes of fecal microbiota transplantation (FMT) combined with atezolizumab plus bevacizumab. **a** Best radiological response, objective response, and disease control of all 12 patients according to modified Response Evaluation Criteria in Solid Tumors (mRECIST) and RECIST version 1.1 (RECISTv1.1). Waterfall plots showing the best radiological response of all 12 patients according to mRECIST (**b**) and RECISTv1.1 (**c**), with colors representing donor group (green, healthy donor FMT; blue, anti-PD-1-responding donor FMT). Progression-free survival (PFS) of all 12 patients according to mRECIST (**d**) and RECISTv1.1 (**e**). **f** Overall survival (OS) of all 12 patients.

Secondary endpoints included radiological response and survival. All 12 patients had at least one follow-up imaging and were therefore evaluable for the best objective radiological response. According to mRECIST, 4 subjects had partial response and 6 patients had stable disease accounting for an overall response rate of 33.3% (95% CI, 9.9%–65.1%) and a disease control rate of 83.3% (95% CI, 51.6%–97.9%); two patients had progressive disease (Fig. 2). Median duration of response was 11.0 (95% CI, not reached—not reached) months. Of the four patients who responded, 2 received FMT from the anti-PD-1-responding donor and 2 received FMT from the healthy donor; three had responded partially to prior atezolizumab plus bevacizumab and one patient had progressive disease as best overall response (Supplementary Table 1).

According to RECISTv1.1, 3 partial responses, 7 stable diseases, and 2 progressive diseases were observed; overall response rate and disease control rate were 25.0% (95% CI, 5.5%–57.2%) and 83.3% (95% CI, 51.6%–97.9%), respectively (Fig. 2). Median duration of response was 11.0 (95% CI, not reached–not reached) months. Two of the 3 responses occurred in patients who received FMT from the anti-PD-1-responding donor. All responders had a partial response to prior atezolizumab plus bevacizumab (Supplementary Table 1).

By the clinical cutoff date, 8 and 9 patients had progressed according to mRECIST and RECISTv1.1, respectively, and 4 patients had died. Median PFS was 6.9 (95% CI, 3.0–10.8) months according to mRECIST and 4.3 (95% CI, 0.2–8.5) months according to RECISTv1.1 (Fig. 2). Median overall survival was 15.5 (95% CI, not reached–not reached) months with a one-year overall survival rate of 69% (Fig. 2).

Notably, a prespecified interim analysis was performed after the first 6 patients had completed at least one radiological follow-up examination, and based on a disease control rate of 83.3%, the decision to continue recruitment in this trial was made.

### Gut microbiome profiling and engraftment after FMT combined with atezolizumab plus bevacizumab

**Fig. 3:**
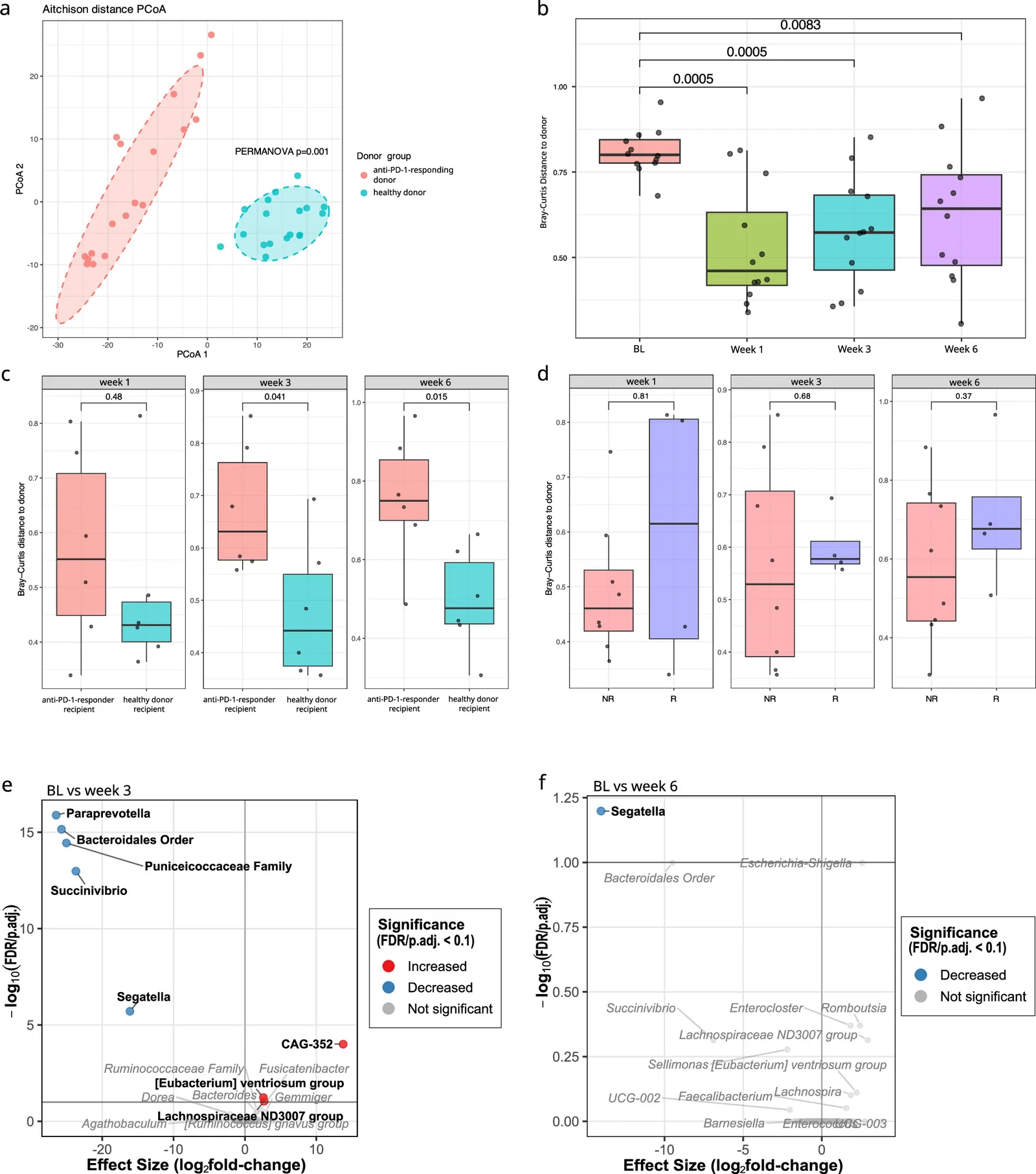
Gut microbiome and fecal metabolites profiling after fecal microbiota transplantation (FMT). **a** Aitchison distance Principal Coordinates Analysis (PCoA) plot of bacterial composition after FMT (weeks 1, 3, and 6) shows clustering for donor groups (anti-PD-1-responding donor group (red), *n* = 6; healthy donor group (turquois), *n* = 6; three time points per patient; PERMANOVA *p* = 0.001). **b** Bray–Curtis distance between recipient and donor of microbiome composition at baseline (BL), weeks 1, 3, and 6 after FMT was calculated to assess changes over time. A reduction in distance indicates greater similarity between donor and recipient composition (*n* = 12; paired Wilcoxon signed-rank test (two-sided)). **c** Bray–Curtis distance between recipient and donor of microbiome composition at week 1, 3, and 6 after FMT to assess similarity over time according to donor group (anti-PD-1-responder recipients, *n* = 6; healthy donor recipients, *n* = 6). A reduction in distance indicates greater similarity between donor and recipient composition (Mann–Whitney *U* test (two-sided)). **d** Bray–Curtis distance between recipient and donor of microbiome composition at week 1, 3, and 6 after FMT to assess similarity over time according to responder status (non-responder (NR), *n* = 8; responder (R), *n* = 4). A reduction in distance indicates greater similarity between donor and recipient composition (Mann–Whitney *U* test (two-sided)). Box plots in panel **b**–**d** show the median (center line), 25th and 75th percentiles (box boundaries), and whiskers extending to the most extreme values within 1.5 × IQR; individual points represent individual observations. Volcano plots illustrating changes of the genus-level fecal microbiome composition at week 3 (**e**) and week 6 (**f**) after FMT compared to baseline (*n* = 12). The *x*-axis represents log₂ fold change, and the y-axis shows –log₁₀ adjusted *p*-values (DESeq2 with paired design controlling for subject-level variation) after Benjamini–Hochberg (BH) correction, with higher values indicating greater statistical significance, significance threshold: false discovery rate (FDR) < 0.1 (two-sided).

Changes in fecal microbial composition were analyzed as an exploratory objective. To determine whether FMT altered recipient gut microbial composition, we assessed β-diversity using Aitchison distance and principal coordinates analysis (PCoA). Post-FMT samples (weeks 1, 3, and 6) clustered according to donor source, demonstrating distinct community structures in recipients of stool from the anti-PD-1-responding donor compared with those receiving stool from the healthy donor (PERMANOVA *p* = 0.001; Fig. 3a; Supplementary Fig. 2a). In contrast, stratification by radiological response did not reveal clear clustering patterns (Supplementary Fig. 2b), indicating that donor source rather than responder status associated with overall microbiome composition.

Donor-recipient similarity was quantified using Bray–Curtis distances. Across the cohort, microbial communities became more similar to their respective donors following FMT, with significant reductions in donor-recipient distance observed at weeks 1, 3, and 6 compared to baseline (Fig. 3b), consistent with donor microbiota engraftment. However, Bray-Curtis dissimilarity to the matched donor increased significantly over weeks 1–6 (linear mixed-effects model: bray ~ week+(1|patient), *p* = 0.020), indicating that the microbiota composition drifted away from that of the donor following the initial effect. Stratification by donor group demonstrated sustained convergence toward donor profiles primarily among recipients of the healthy donor FMT (Fig. 3c). When analyzed by radiological response status, no significant differences in donor–recipient similarity were detected between responders (R) and non-responders (NR) (Fig. 3d).

Within-sample microbial diversity (α-diversity) was assessed to determine whether FMT altered overall community complexity. Baseline α-diversity varied substantially across individuals. Longitudinal profiling revealed dynamic post-transplant changes with donor-dependent trajectories (Supplementary Fig. 2c, d). Recipients of stool from the healthy donor generally exhibited higher Shannon diversity across post-FMT time points, whereas recipients of stool from the anti-PD-1-responding donor showed more variable or modest changes. Individual-level trajectories indicated that diversity shifts occurred rapidly after transplantation and stabilized by weeks 3-6. Post FMT dynamics of Shannon diversity showed a significant donor-group-dependent trajectory (linear mixed effects model: Shannon ~ week × donor_group+(1|patient)): recipients of healthy donor FMT exhibited a significant increase in diversity over weeks 1–6 (*p* = 0.0006), whereas recipients of anti-PD-1 responder FMT showed no temporal change (*p* = 0.93). Together, these findings demonstrate donor-dependent restructuring of within-sample microbial diversity following FMT.

FMT was associated with significant alterations in stool microbial composition at the genus level (Fig. 3e, f), with the most pronounced changes observed at week 3 after FMT. The relative abundance of CAG‑352 (uncultured Ruminococcaceae), [Eubacterium] ventriosum group, and Lachnospiraceae ND3007 group increased at week 3 compared to baseline, while that of Segatella, Succinivibrio, Puniceicoccaceae family, Bacteroidales order, and Paraprevotella decreased (Fig. 3e). Distinct patterns were also observed between responders and non-responders (Supplementary Fig. 2e, f).

### Changes in fecal and plasma bile acid profiles after FMT combined with atezolizumab plus bevacizumab

Since gut microbes participate in bile acid metabolism by the formation of secondary bile acids, we profiled fecal and plasma bile acids longitudinally by reversed-phase LC–MS/MS (liquid chromatography tandem mass spectrometry) as an exploratory objective. FMT was associated with a limited change in fecal and plasma secondary bile acid profiles (Supplementary Fig. 3a–d). Both fecal ursodeoxycholic acid (UDCA) and plasma isoursodeoxycholic acid (isoUDCA) levels significantly decrease at week 6 compared to baseline (Supplementary Fig. 3b, d). When comparing responders and non-responders, 3-oxo-chenodeoxycholic acid (X3.oxo.CDCA) was enriched in the stool of responders at week 3 and 6, and plasma deoxycholic acid 3-sulfate was enriched in non-responders at week 3 (Supplementary Fig. 3e–g).

### Changes in peripheral blood mononuclear cells after FMT combined with atezolizumab plus bevacizumab

**Fig. 4:**
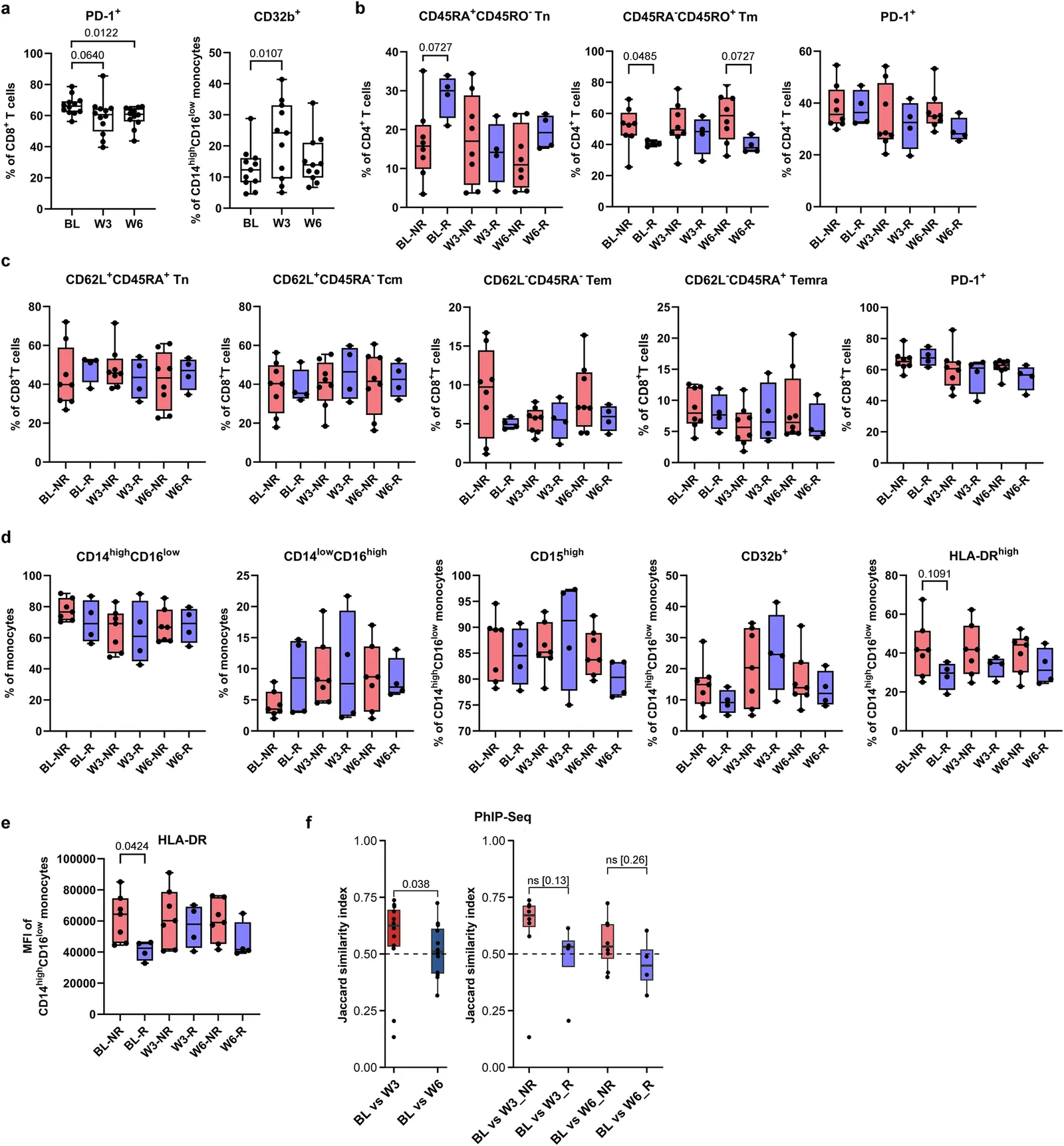
Immune changes after FMT combined with atezolizumab plus bevacizumab assessed by peripheral blood mononuclear cells (PBMC) analysis and phage immunoprecipitation sequencing (PhIP-Seq). **a** Percentage of PD-1^+^CD8^+^ T cells (left, *n* = 12) and CD32b^+^CD14^high^CD16^low^ monocytes (right, *n* = 11) at different time points (BL, baseline; w3, week 3; w6, week 6). Paired Wilcoxon signed rank test (two-sided). **b** Percentage of naïve (Tn) and memory T (Tm) cells as well as PD-1^+^ cells in the CD3^+^CD4^+^ T cell fraction in non-responders (NR, *n* = 8) and responders (R, *n* = 4) at BL, W3, and W6. Mann–Whitney *U* test (two-sided). **c** Percentage of naïve (Tn), central memory (Tcm), effector memory (Tem), effector memory RA^+^ (Temra) T cells, and PD-1^+^ cells in the CD3^+^CD8^+^ T cell fraction in NR (*n* = 8) and R (n = 4) at BL, W3, and W6. Mann–Whitney *U* test (two-sided). **d** Percentage of CD14^high^CD16^low^ and CD14^low^CD16^high^ cells within the monocyte gate and percentage of CD15^+^, CD32b^+^ and HLA-DR^high^ within the CD14^high^CD16^low^ fraction in NR (*n* = 7) and R (*n* = 4) at BL, W3 and W6. Mann–Whitney *U* test (two-sided). **e** Mean fluorescence intensity (MFI) of HLA-DR on CD14^high^CD16^low^ monocytes in NR (*n* = 7) and R (*n* = 4) at BL, W3, and W6. Mann–Whitney *U* test (two-sided). Box plots in panels **a**–**e** show the median (center line), 25th and 75th percentiles (box boundaries), and minimum and maximum values (whiskers); individual points represent individual observations. **f** Longitudinal stability of antibody epitope repertoires measured by PhIP-Seq. Box plots show the distribution of within-individual Jaccard similarity indices, quantifying the overlap of detected antibody-binding peptides between BL and follow-up time points. Left, pairwise temporal comparisons between BL and W3 or W6 (*n* = 12). Right, the same comparisons stratified by radiological response (R, *n* = 4; NR, *n* = 8). The dashed line marks a Jaccard similarity of 0.5. Statistical significance was assessed using aligned rank transform (ART) models; adjusted *p*-values (two-sided) are shown, with non-significant comparisons indicated as ns. Box plots in panel **f** show the median (center line), 25th and 75th percentiles (box boundaries), and whiskers extending to the most extreme values within 1.5×IQR; individual points represent individual observations.

Changes in peripheral blood mononuclear cells, measured by flow cytometry, were assessed as an exploratory objective at baseline and week 3 and 6 after FMT (Fig. 4a–e). One baseline sample for monocyte analysis was unavailable from a single patient, and consequently, this patient was excluded from the respective analysis. The proportion of PD-1^+^CD8^+^ T cells decreased at week 3 and 6 compared to baseline (before FMT), while the proportion of CD32b^+^CD14^high^CD16^low^ monocytes significantly increased at week 3 and decreased almost back to baseline levels at week 6 (Fig. 4a). The proportion of memory T cells (CD45RA^−^CD45RO^+^) within the CD3⁺CD4⁺ T cell compartment was higher in non-responders compared to responders at baseline (Fig. 4b). Similarly, mean fluorescence intensity (MFI) of HLA-DR on CD14^high^CD16^low^ monocytes was higher in non-responders than in responders at baseline (Fig. 4e). Other lymphocyte populations of CD4+ and CD8+ T cells (Fig. 4b, c), as well as several monocyte populations (Fig. 4d) remained relatively stable over time and were not significantly different between responders and non-responders at different time points.

### Longitudinal stability of antibody epitope repertoires following FMT combined with atezolizumab plus bevacizumab

To assess temporal dynamics of systemic humoral immunity as an exploratory analysis, we quantified longitudinal changes in IgG antibody epitope repertoires using phage immunoprecipitation sequencing (PhIP-Seq) (Supplementary Fig. 4). Across all patients, antibody repertoires exhibited significantly higher similarity between BL and W3 than between BL and W6 (Fig. 4f; Supplementary Fig. 5), indicating progressive divergence of humoral responses over the course of treatment.

Stratification by radiological response revealed distinct early temporal patterns. Non-responders showed higher intra-individual similarity between BL and W3, consistent with the relative stability of antibody repertoires three weeks after FMT. In contrast, responders displayed reduced similarity between BL and W3, reflecting a more pronounced early remodeling of systemic antibody reactivity. A similar trend was observed between BL and W6 in both groups, consistent with increased repertoire divergence at later time points (Fig. 4f).

Importantly, these longitudinal differences in stability were not accompanied by major changes in overall antibody repertoire size, diversity, or global composition. Alpha diversity measures, including the number of enriched peptides and Shannon diversity at both peptide and species levels, remained largely stable across time points and response groups (Supplementary Fig. 6a–d). Likewise, beta diversity analyses based on Bray-Curtis dissimilarity revealed no significant global separation of antibody repertoires by time point or response status (Supplementary Fig. 6e–h).

## Discussion

In this single-center, open-label, single-arm phase II pilot study, FMT combined with atezolizumab plus bevacizumab was feasible and demonstrated a manageable safety profile, consistent with known adverse events of the respective interventions^9,20^. We observed a favorable ORR according to mRECIST and RECISTv1.1 of 33% and 25%, respectively, which is similar to response rates reported in patients with HCC receiving first-line immunotherapy^3,5,9^, and higher than those observed in patients with different solid tumors treated with immunotherapy beyond progression^21,22^. The DCR according to both mRECIST and RECISTv1.1 was 83%, indicating that 10 patients experienced a treatment benefit, while only two patients had progressive disease at the first radiological evaluation.

Following FMT, recipient microbiome composition appeared more similar to that of the donor, and healthy donor FMT seemed to be associated with higher engraftment compared with anti-PD-1-responder FMT. After FMT, the microbiome showed a compositional pattern suggestive of improved immunotherapy responsiveness, with enrichment of certain taxa (i.e., CAG‑352 (uncultured Ruminococcaceae), [Eubacterium] ventriosum group) associated with immunotherapy response^23,24^, and a reduction of taxa that have been linked to non-response (i.e., Bacteroidales order)^10^. Moreover, the levels of the secondary bile acids UDCA, which has been linked to peripheral immunosuppression^25,26^, and its derivative isoUDCA appeared to be decreased following FMT in stool and plasma, respectively.

We also observed longitudinal systemic immune changes, including a decrease in the circulating PD-1^+^CD8^+^ T cell population, which may reflect redistribution into tumor tissue or a downregulation of PD-1 upon activation. The early increase in circulating CD32b^+^CD14^high^CD16^low^ monocytes may be consistent with counterregulatory mechanisms following immune activation. Comparing responders versus non-responders at different time points, we found a higher proportion of peripheral CD4^+^ memory T cells and an increased expression of HLA-DR on CD14^high^CD16^low^ monocytes in non-responders at baseline. In the context of prior exposure and refractoriness to immunotherapy, these findings may be consistent with a more inflamed state, but with reduced potential for effective antitumor immunity. Finally, PhIP-Seq to profile systemic IgG-epitope repertoires against gut microbiota and environmental antigens revealed that antibody repertoire stability, rather than global diversity or composition, changed over time, and this finding was more pronounced in responders compared to non-responders. This qualitative remodeling of the antibody repertoire may reflect exposure to novel microbial antigens following FMT. Importantly, all exploratory analyses should be considered hypothesis-generating and interpreted with caution.

While several studies have investigated FMT combined with immunotherapy in patients with melanoma, lung cancer, renal cell carcinoma, and colorectal cancer in the first-line setting or in immunotherapy-naïve patients^27–31^, the evidence of this approach in immunotherapy-refractory cancers is limited to small pilot studies in melanoma^14,15^ and different gastrointestinal cancers^16^. Our study differs in several key aspects from the latter three studies. Firstly, our cohort was more homogenous since all patients had the same cancer type (i.e., hepatocellular carcinoma), progressed on the same prior immunotherapy administered as systemic first-line (i.e., atezolizumab plus bevacizumab), and were then retreated, together with FMT, with the same ICI-based regimen on which they had progressed (i.e., atezolizumab plus bevacizumab). In contrast, other trials included patients with different cancer types who had received prior immunotherapy only in later-line settings^16^, or even when the prior immunotherapy regimen differed from the ICI administered after FMT^14,15^. This is of particular importance, since rechallenge with a different ICI regimen alone may be sufficient to induce a response in immunotherapy-refractory cancers^32,33^. Secondly, all three studies relied exclusively on responder-derived FMT^14–16^, while half of our patients had responder-derived FMT and the other half received FMT from a healthy donor. In fact, the latter resulted from a protocol amendment following the publication of a phase I trial demonstrating the safety of healthy donor FMT in combination with first-line immunotherapy in patients with advanced melanoma^27^. Given the difficulties of collecting donor stool from patients with HCC and durable response to immunotherapy, who are rarely found in clinical practice and often have significant comorbidities, the use of healthy donors represents a more practical alternative.

Notably, 2 patients achieved a response in each FMT donor group (anti-PD-1-responding and healthy donor). Thus, we hypothesize that the responses may not have been donor-specific, but rather related to FMT itself, potentially in part through immune stimulation induced by the introduction of new microbes and a shift toward a microbial composition favoring immunotherapy responsiveness.

We want to acknowledge some limitations. Given the limited sample size and the lack of a control group, the clinical findings of this study remain preliminary and warrant cautious interpretation. The inclusion of patients who received other treatments after progressing on atezolizumab plus bevacizumab and before study inclusion may have introduced a potential confounding factor. The results from exploratory analyses (i.e., fecal microbiome analyses, bile acid profiling, immunophenotyping of immune cells, PhIP sequencing) are primarily descriptive and should therefore be considered hypothesis-generating. Moreover, although inclusion of multiple donors is common in FMT trials, the use of 2 donors with distinct clinical characteristics (one older female anti-PD-1 responder; one younger healthy male) could have introduced additional biological variability.

In conclusion, atezolizumab plus bevacizumab combined with a single FMT administered via colonoscopy was safe and appeared effective in overcoming resistance in a subset of patients with HCC who progressed during prior treatment with atezolizumab plus bevacizumab. FMT, together with continuation of immunotherapy, could be a treatment option, particularly for patients with minor progression who had experienced a prior response to immunotherapy. However, confirmation of these results in larger prospective trials is required.

## Methods

### Study design and participants

This single-center, open-label, single-arm, phase II pilot study (ClinicalTrials.gov identifier: NCT05750030) was conducted at the Medical University of Vienna in Austria. The protocol of this study has been published previously^34^. Between August 2023 and November 2024, patients with advanced HCC who previously progressed on atezolizumab plus bevacizumab were included. Patients received one FMT via colonoscopy plus regular interval atezolizumab plus bevacizumab. The study was approved by the Ethics Committee of the Medical University of Vienna (1054/2022) in accordance with applicable Austrian regulations and the requirements of the Austrian Federal Office for Safety in Health Care (BASG). The study was performed in full compliance with the legal regulations of the Austrian Medicinal Products Act (AMG–Arzneimittelgesetz), and was registered in the European Clinical Trial Database (EudraCT number: 2022-000234-42) and the European Union Clinical Trials Information System (CTIS number: 2024-516835-29-00). Two relevant amendments were disclosed to and approved by all respective authorities during the conduct of this study: a substantial amendment (change of in-/exclusion criteria) to also allow inclusion of patients with a prior response before progressing on atezolizumab plus bevacizumab, and a non-substantial amendment to include a healthy donor. A list of all protocol amendments is provided in Supplementary Table 3. The conduct of the study complied with the Declaration of Helsinki and the principles of Good Clinical Practice. All patients provided written informed consent and were able to withdraw their consent at any given timepoint. Study participants received no compensation. The Clinical Trials Coordination Center (KKS) of the Medical University of Vienna monitored the conduct of the trial and provided administrative support.

### Sex and gender considerations

Biological sex was recorded for all participants at enrollment. Gender identity was not recorded. Sex was not used as an inclusion or exclusion criterion. Since the study was not powered to evaluate sex-based differences, no analyses stratified by sex were performed.

### Patient eligibility criteria

*Inclusion criteria*

- Patients must be 18 years or older and have histologically or radiologically confirmed HCC.
- Patients must have progressed (according to mRECIST) on previous treatment with atezolizumab plus bevacizumab (with or without prior complete or partial response) OR must have had stable disease as best radiological response (according to mRECIST) after the first 12 months of treatment with atezolizumab plus bevacizumab.
- Patients must have a negative HIV test.
- Patients with chronic hepatitis B must be under antiviral treatment and hepatitis B DNA must be <500 IU/mL.
- Variceal status must be known, and if present, adequate medical or endoscopic treatment for varices is required.
- Patients with ECOG performance status 0–1.
- Patients with Child-Pugh class A-B8.
- Patients must have adequate hematological and end-organ functions.
- Women of childbearing potential must agree to remain abstinent (refrain from heterosexual intercourse) or use adequate contraceptive methods.
- Men must agree to remain abstinent (refrain from heterosexual intercourse) or use a condom.

*Exclusion criteria*

- Known fibrolamellar carcinoma or mixed hepatocellular-cholangiocellular carcinoma.
- Massive tumor progression (>100% increase in target lesions or progression associated with significant clinical deterioration).
- Uncontrolled ascites and/or overt hepatic encephalopathy or concomitant treatment with rifaximin.
- Prior allogeneic stem cell or solid organ transplantation.
- Active or history of severe autoimmune disease.
- History of idiopathic pulmonary fibrosis, organizing pneumonia (e.g., bronchiolitis obliterans), drug-induced pneumonitis, or idiopathic pneumonitis, or evidence of active pneumonitis.
- Significant cardiovascular disease (such as New York Heart Association Class II or greater cardiac disease, myocardial infarction, or cerebrovascular accident) within 3 months prior to study inclusion or unstable angina.
- Severe infection within 4 weeks prior to study inclusion.
- Women who are pregnant or breastfeeding.
- Ongoing systemic immunosuppressive medication with the following exceptions: Acute, low-dose systemic immunosuppressant medication or a one-time pulse dose of systemic immunosuppressant medication (e.g., 48 h of corticosteroids for contrast allergy), and mineralocorticoids (e.g., fludrocortisone), corticosteroids for chronic obstructive pulmonary disease or asthma, or low-dose corticosteroids for adrenal insufficiency.
- Significant vascular disease (e.g., peripheral arterial thrombosis) within 6 months prior to study inclusion.
- Major surgery within 4 weeks prior to study inclusion or minor surgery (excluding placement of a vascular access device) within 3 days prior to study inclusion.
- History of gastrointestinal fistula, perforation, or intra-abdominal abscess within 6 months prior to study inclusion.
- Serious, non-healing wound or active ulcer disease.

### Trial sample size

As this study was designed as a pilot study evaluating safety and feasibility, and because of its single-arm design, no formal sample size calculation was performed. The predefined sample size of this trial was 12 patients. The rationale was that at the time this study was designed, no comparable studies in HCC had been published, and the available evidence was limited to two studies evaluating the safety and efficacy of FMT in immunotherapy-refractory patients with advanced melanoma, which included a comparable sample size (*n* = 10 and 16)^14,15^. Thus, the number of patients was expected to sufficiently fulfill the pilot study’s main objectives. No inferential statistical testing was used for the calculation of primary safety and secondary efficacy endpoints.

### FMT donor selection criteria

The FAB-HCC trial included two donors, of whom one was a patient with HCC who responded to anti-PD-1 treatment (donor 1) and one was a healthy individual (donor 2). Clinical characteristics of the two FMT donors, which were included in June 2022 and November 2023, are displayed in Supplementary Table 2. Prior to fecal material collection and preparation, donors and donor material were thoroughly screened, and infectious agents were ruled out by repetitive stool sampling. In- and exclusion criteria for donors are listed below.

*Donor-specific inclusion criteria*

- Patients with unresectable HCC treated with anti-PD-(L)1-based immunotherapy with a complete or partial response for at least 12 months.
- Healthy donors.

*Donor-specific exclusion criteria*

- History of antibiotic treatment within 2 months prior to stool donation.
- Known present or past intrinsic gastrointestinal illnesses, including: inflammatory bowel disease, irritable bowel syndrome, chronic diarrhea (i.e., celiac disease), active primary gastrointestinal malignancies, or major gastrointestinal surgical procedures.
- Known symptomatic autoimmune illness.
- History of documented chronic pain syndromes (i.e., fibromyalgia, chronic fatigue) or neurologic and neurodevelopmental disorders.
- History of metabolic syndrome, severe obesity (body mass index > 35), or moderate-to-severe malnutrition.
- Positivity for SARS-CoV-2, latent potential infections (human immunodeficiency virus, hepatitis B virus, hepatitis C virus [only if PCR positive], human T-cell lymphotropic virus type 1 [HTLV-1], HTLV-2, strongyloides, syphilis), and/or evidence of multi-drug-resistant organisms (e.g., vancomycin-resistant Enterococcus, carbapenem-resistant Enterobacteriaceae, extended spectrum beta-lactamase).
- Evidence of FMT-relevant severe immune-related adverse events (i.e., colitis, hepatitis).

### Preparation of fecal material for FMT

Fecal material was collected and processed according to standardized protocols^35,36^. Donor feces were collected and processed after the completion of the safety screening phase. For the preparation process, regulations for the work with feces, classified as biohazard level 2, were followed (i.e., wearing of water-repellent garments, gloves, facemasks, protective goggles, or shields). After the extraction of the fecal probe (~50 g of fecal matter for one colonoscopy-based implant), the entire donation was diluted with 100–500 mL of sterile saline (0.9% NaCl) using 3× the weight of donor stool. Afterward, the mixture was homogenized in a sterilized blender or similar device, especially designated for this purpose. Afterward, the probe was sequentially sieved to remove particulate material. The final slurry was concentrated 3-fold by centrifugation.

Since instant application of fresh fecal probes to study recipients was not possible, the processed donor stool was mixed with glycerol (10% of total weight) and frozen at a monitored temperature (−80 °C). Before usage, the fecal probes were gently reheated in a 37 °C warm water bath over a period of 2 h.

### Fecal microbiota transplantation

FMT via colonoscopy was performed according to the Austrian consensus^37^ and the European guidelines on FMT^38^. After a gut microbiota depletion phase, where patients received the oral antibiotic regimen consisting of vancomycin 500 mg and neomycin 1000 mg q6h for 72 h, patients were prepared with a colon lavage according to standard care. Colonoscopy was performed by an expert endoscopist who administered a total of 100 mL fecal implant suspension via a catheter (20 mL in the terminal ileum, 40 mL in the cecum and right colon, 20 mL in the transverse colon, and 20 mL in the descending colon). After completion of FMT, which was considered the baseline of the study, patients remained lying in the prone Trendelenburg position for 4 h to maintain the fecal suspension in the bowel.

### Treatment with atezolizumab plus bevacizumab

Patients included in this study received the investigational medicinal product (IMP) atezolizumab (manufacturer: Roche), a monoclonal antibody against programmed death-ligand 1 (PD-L1), in combinations with bevacizumab (manufacturer: Roche), a monoclonal antibody against vascular endothelial growth factor (VEGF), at the approved standard doses^9^. Atezolizumab 1200 mg and bevacizumab 15 mg/kg were administered by intravenous infusion on day 1 following FMT (±3 days) and every 21 days (±3 days) thereafter, until loss of clinical benefit or unmanageable toxicity.

### Assessments and definitions

First tumor assessment by contrast-enhanced computed tomography of chest and abdomen was performed at week 6, followed every 12 weeks thereafter. Radiological response was assessed by an expert radiologist according to mRECIST^39^ and RECIST v1.1^40^. Objective response rate (ORR) was defined as the number of patients with a complete (CR) and partial response (PR), and designated as responders (R) for exploratory analyses. Patients with stable disease (SD) and progressive disease (PD) as best response were designated as non-responders (NR). Disease control rate (DCR) was defined as the number of patients with CR, PR, and SD. Duration of response (DoR) was calculated from the date of first radiological response until the date of first radiological progression; patients who discontinued from the study without radiological progression were censored at the date of the last study-specific imaging. Progression-free survival (PFS) was defined as the time from FMT to first radiological progression or death, whichever came first; patients who discontinued from the study without radiological progression and who were still alive 12 weeks after the last study-specific imaging were censored at the date of the last study-specific imaging. Overall survival (OS) was calculated from the date of FMT until the date of death; patients who were still alive were censored at the clinical cutoff date.

A prespecified interim analysis was performed after the first 6 patients had completed at least one radiological follow-up examination. To ensure that a likely unsuccessful therapeutic strategy was not pursued, enrollment would have been stopped prematurely if the disease control rate among the first 6 patients included was below 50%. The end of the study was defined as the date when the last patient enrolled had a follow-up of at least 20 weeks, if not progressed or deceased earlier.

### Data and sample collection

At each clinical visit, patient data obtained from a complete clinical assessment and laboratory work-up were collected and entered in the electronic data capturing system ‘clincase’ (Version 2.7.0.12, Quadratek Data Solutions Ltd.). Access to the database was restricted to the study investigators, delegated study personnel, and the study monitors. Patient blood and stool sampling for exploratory analyses took place before FMT at screening and at several timepoints thereafter.

Stool samples were collected using a kit composed of a sterile 30 mL stool container, a sampling diaper, and a cooler bag. Patients were requested to sample their own stool at home, up to 24 h before their arrival at the clinic. The containers were frozen in the patients’ home freezer (−20 °C) and transported to the clinic in a cooler bag. Upon receipt, the containers were frozen in a −80 °C temperature-monitored freezer.

### Adverse events assessment and management

All adverse events (AEs), whether reported by the patient or noted by study personnel, were evaluated according to the National Cancer Institute Common Terminology Criteria of Adverse Events, version 5.0 (CTCAE v5.0), recorded, and classified as FMT-related, IMP-related, or IMP-unrelated. To ensure consistency in AE designation and grading, the principal investigator reviewed all recorded adverse events. Management of AEs in accordance with routine clinical practice guidelines was performed by the treating physicians. IMP was interrupted or discontinued at the discretion of the treating physician.

### Outcomes

The primary objective of this study was safety as measured by the incidence and severity of treatment-related adverse events. Secondary objectives included assessment of best radiological response, ORR, DCR, PFS, and OS. Exploratory objectives included changes in fecal microbiome composition, changes in PBMCs, changes in fecal and plasma bile acid metabolites, and changes in the systemic IgG-epitope repertoires against gut microbiota and environmental antigens.

### Statistical analysis of clinical data

Statistical analyses were performed using IBM SPSS Statistics 30 (SPSS Inc., Armonk, New York, USA), R 4.3.1 (R Core Team, R Foundation for Statistical Computing, Vienna, Austria), and GraphPad Prism 9 (GraphPad Software, La Jolla, California, USA). Continuous variables were reported as mean ± standard deviation (SD) or median (IQR), and categorical variables were shown as numbers (*n*) and proportions (%) of patients. Exact (Clopper-Pearson) 95% confidence intervals for ORR and DCR were computed in R using the package “contingencytables” (https://www.rdocumentation.org/packages/contingencytables/versions/3.1.0/topics/ClopperPearson_exact_CI_1x2). Median estimated follow-up was calculated by the reverse Kaplan–Meier method. Survival curves were created using the Kaplan–Meier method. No data were excluded from the analyses.

### Gut microbiota analysis

DNA extraction, 16S rRNA gene sequencing, and raw data processing were performed at the Joint Microbiome Facility of the Medical University of Vienna and the University of Vienna (project ID JMF-2401-16) using a two-step PCR dual-barcoding workflow optimized for high-throughput sequencing^41^. Amplicon pools were extracted from the raw sequencing data using the FASTQ workflow in BaseSpace (Illumina) with default parameters. Demultiplexing was performed with the Python package demultiplex (Laros JFJ, github.com/jfjlaros/demultiplex), allowing one mismatch for barcodes and two mismatches for linkers and primers. Amplicon sequence variants (ASVs) were inferred using the DADA2 R package v1.35.8^42^, applying the recommended workflow^43^. FASTQ reads 1 and 2 were trimmed at 220 nt and 150 nt with allowed expected errors of 2, respectively. ASV sequences were subsequently classified using DADA2 and the SILVA database SSU Ref NR 99 release 138.2^44,45^ using a confidence threshold of 0.5. ASVs without classification or classified as mitochondria or chloroplasts were removed. After filtering, only samples yielding at least 1000 read pairs were kept for further analyses.

Downstream analyses were performed with R version 4.4.2 using vegan (v2.6-8)^46^, phyloseq (v1.50.0)^47^, and DESeq2 (v1.46.0)^48^. Aitchison distances were calculated from (clr+1) transformed ASV counts and analyzed by PERMANOVA (adonis2)^46^. Bray–Curtis distances between donor and recipient were calculated using phyloseq and total sum scaled ASV tables. Differential abundance was assessed at the genus level using DESeq2 with paired design (~subject + timepoint), local dispersion fitting, and Wald test. Statistical significance was defined by a false discovery rate (FDR) of <0.1 (two-sided) after Benjamini–Hochberg correction; effect sizes are log_2_ fold-changes. Linear discriminant analysis effect size (LEfSe) was performed to identify bacterial genera that were differentially abundant between response groups at week 3 and 6, using the Kruskal–Wallis test (*α* = 0.05, two-sided) for statistical significance. Post-FMT microbiome dynamics were assessed using linear mixed-effects models fitted with the R package lme4/lmerTest^49^. We used the week as a continuous fixed effect and patient as a random intercept.

### Targeted mass spectrometry

Bile acids (BAs) and their conjugates were measured with reversed-phase liquid chromatography–tandem mass spectrometry (LC–MS/MS). Retention times, SRM-transitions, and optimal collisional energies were determined by authentic standards. All data interpretation was performed using Xcalibur (Thermo Fisher Scientific). In brief, 1 μL of the extract was injected on a Kinetex C8 column (100 Å, 100 × 2.1 mm; Phenomenex) with the respective guard column, employing a flow rate of 100 μL/min. A 21-min-long gradient from 80% A (2.5 mM ammonium acetate in water, 0.1% acetic acid) to 90% B (2.5 mM ammonium acetate in methanol, 0.1% acetic acid) was used for the separation. The HPLC (RSLC Ultimate 3000; Thermo Fisher Scientific) was directly coupled to a TSQ Altis mass spectrometer (Thermo Fisher Scientific) via electrospray ionization. BAs and conjugates were analyzed in the negative ion mode, employing the respective transitions (e.g., taurocholic acid *m*/*z* 514 to *m*/*z* 80) employing optimized collision energies as determined by fragmenting authentic standards, which have also been used for validating retention times by standard addition to a pooled quality control sample.

Bile acid analysis (stool and plasma) was performed in R version 4.4.2. Metabolites were filtered (prevalence ≥ 5%, variance > 25th percentile) and normalized by total sum scaling. Differences between baseline and post-FMT timepoints (week 3, week 6) were assessed using paired Wilcoxon signed rank tests. Statistical significance was defined by an FDR of <0.1 (two-sided) after Benjamini–Hochberg correction; effect sizes are median differences in normalized abundances. Linear discriminant analysis effect size (LEfSe) was performed to identify metabolites differentially abundant between response groups at week 3 and 6, using the Kruskal–Wallis test (*α* = 0.05, two-sided) for statistical significance.

### Immunophenotyping of peripheral blood mononuclear cells (PBMCs)

Aliquots of cryopreserved PBMCs (1 × 10^7^ cells/mL) were thawed in RPMI 1640 supplemented with 10% FBS, washed twice, and resuspended in a buffer (PBS containing 0.5% FBS and 0.05% NaN3; FACS buffer) (Merck Millipore, Burlington, MA, USA). 1 × 10^6^ cells were incubated with Human TruStain FcX™ Fc Receptor Blocking Solution (Biolegend, San Diego, CA, USA) for 10 min and then stained with a multicolor antibody cocktail covering T cell markers or monocyte markers (Supplementary Table 4) for 30 min at 4 °C. Then, cells were washed twice in FACS buffer and transferred to a 96-well plate for analysis on a CytoFlex LX flow cytometer (Beckman Coulter, Brea, CA, USA) using CytExpert Software V2.5 (Beckman Coulter) for acquisition at the Core Facility Flow Cytometry of the Medical University of Vienna. Subsequently, T cell and monocyte phenotypes were analyzed using FlowJo V10.10.0 software (Beckton Dickinson, Franklin Lakes, NJ, USA). The gating strategy for T cells and monocytes is provided in Supplementary Fig. 7. Graphical illustrations and statistical comparisons were performed by means of GraphPad Prism 11 (GraphPad Software, La Jolla, CA, USA).

### Phage immunoprecipitation sequencing

Three antigen libraries covering a total of 357,000 peptide sequences were used in this study. The first library (244,000 peptides, derived from 28,668 proteins) is primarily focused on gut microbiota antigens, including pathogenic (~10%), probiotic bacteria (~6%)^50^, and antibody-coated bacteria (~9%)^51^, as well as commensal bacteria identified in metagenomic studies of nearly 1000 healthy individuals (~60%)^52^. It also contains virulence factors (~10%) from the Virulence Factor Database^53^ and a set of controls (~5%)^54^. The second library (100,000 variants) comprises antigens from phages (~40%), environmental (~30%) sources^55^, as well as the complete set of B cell epitopes (~40%) from the Immune Epitope Database^56^. The third library (13,000 variants) was created following the COVID-19 pandemic and contains antigens from both seasonal/common cold human and animal coronaviruses^57^. Antigens were rationally selected by prioritizing previously reported bacterial antigens and strains that are known to be recognized by the immune system, as well as uncharacterized antigens. Detailed protocols for library design, cloning, and sequencing have been published^58,59^.

Processing of raw PhIP-Seq data and determination of peptide enrichment were performed largely as described previously^54^, with minor modifications. Single-end 75-nt Illumina sequencing was performed for all plasma samples as well as for the input library, defined as sequencing of the antigen library prior to immunoprecipitation. Raw sequencing reads spanning the peptide-identifying barcode (75 nt) were aligned to a reference database of barcode sequences corresponding to expressed peptides using Bowtie2^60^, allowing up to two mismatches. Reads mapping to each barcode were quantified using SAMtools^61^ and *awk*. Enriched clones were identified by fitting a three-parameter zero-inflated generalized Poisson (ZIGP) distribution^62^ to read counts from clones sharing identical input library counts. Prior to parameter estimation, the top 1% of read counts within each input count bin were excluded to reduce the influence of extreme values. ZIGP parameters were modeled as functions of input read count by fitting cubic splines constrained to be strictly increasing for parameters *λ* and *θ*, and strictly decreasing for parameter *π*, using the scam package in R. Peptides were considered significantly enriched if their observed read counts corresponded to a *p*-value ≤ 0.05 after Holm correction for multiple testing.

For downstream analyses, antibody epitope repertoires were represented as binary matrices indicating the presence or absence of enriched peptides for each sample, as defined by the enrichment procedure described above.

The number of enriched peptides was calculated per sample as a measure of repertoire size. Shannon diversity indices were computed from the binary enrichment data at both the peptide level and the species level, where peptides were aggregated according to their taxonomic annotation. To assess global similarity of antibody repertoires across samples, Bray-Curtis dissimilarities were computed from relative species abundance matrices using the vegdist function of the vegan R package (v.2.7.1). Principal coordinate analysis (PCoA) was performed on the resulting distance matrices. Group-level differences in repertoire composition were tested using permutational multivariate analysis of variance (PERMANOVA) implemented with the adonis2 function (1000 permutations). Homogeneity of multivariate dispersion was assessed using the betadisper function followed by permutation testing (permutest, 1000 permutations). Distances to group centroids in PCoA space were extracted to quantify within-group dispersion.

Longitudinal stability of antibody epitope repertoires was quantified using the Jaccard similarity index using the binary peptide enrichment data. Pairwise Jaccard distances were computed using vegdist (method = “jaccard”), and transformed to similarity values as 1−distance. Within-patient similarity values were extracted by comparing samples collected from the same individual at different time points (baseline vs. week 3 and baseline vs. week 6). These within-individual similarity values were summarized using boxplots and, where indicated, stratified by radiological response. Differences in Jaccard similarity indices were assessed using aligned rank transform (ART) models implemented in the ARTool R package, allowing non-parametric testing of factorial effects involving time point and radiological response. Post hoc contrasts were computed using art.con. For comparisons stratified by response, *p*-values were adjusted using the Benjamini–Hochberg procedure.

All analyses were performed in R (v4.5.1) on Ubuntu 24.04.3 LTS. All data visualizations were generated using ggplot2 (v.3.5.2). All figures were generated using the R programming language and BioRender (https://BioRender.com).

### Reporting summary

Further information on research design is available in the Nature Portfolio Reporting Summary linked to this article.

## Supplementary information

Supplementary Information

Reporting Summary

Transparent Peer Review file

## Data Availability

The data generated and analyzed during this study are available within the article, its supplementary information, and publicly accessible repositories. The minimum dataset used for the primary, secondary, and exploratory analyses may be shared with qualified medical or scientific professionals for a defined research purpose. Deidentified individual participant data may be provided after a data sharing agreement has been signed. All requests for data access should be sent to the corresponding author (M.P.). Requests will be considered and responded to within 2 months of receipt. Patient-related data not included in the paper were generated as part of a clinical trial and are subject to patient confidentiality. The 16S rRNA gene amplicon sequencing data have been deposited at the National Center for Biotechnology Information (NCBI) Sequence Read Archive, BioProject ID PRJNA1444467. The PhIP-Seq data and associated metadata used in this study are publicly available in the FAB-HCC repository at https://github.com/csReynaB/FAB-HCC. A permanent archived version of the repository associated with this study is available through Zenodo at https://doi.org/10.5281/zenodo.21921459^63^. The targeted metabolomics data for this study have been deposited in the MetaboLights^64^ repository of the European Bioinformatics Institute (EMBL-EBI) with the dataset identifier MTBLS12023, available at https://www.ebi.ac.uk/metabolights/MTBLS12023.

## Code availability

The code used for PhIP-Seq data processing, statistical analysis, visualization, and modeling in this study is publicly available in the FAB-HCC GitHub repository at https://github.com/csReynaB/FAB-HCC. The version of the code used for this study has been permanently archived on Zenodo and is available at https://doi.org/10.5281/zenodo.21921459^63^.
