# Supplementary material for: FMT combined with atezolizumab-bevacizumab in hepatocellular carcinoma refractory to immunotherapy: an open label, single-arm, phase II pilot study
Source: Nat Commun. 2026 Oct 7;17:11442. doi: 10.1038/s41467-026-78219-z (PMC13736962; doi:10.1038/s41467-026-78219-z)
Supplement: Supplementary file 1 [file 41467_2026_78219_MOESM1_ESM.pdf]

Supplementary Information

FMT combined with atezolizumab-bevacizumab in hepatocellular carcinoma refractory to immunotherapy: an open label, single-arm, phase II pilot study

Katharina Pomej[1,2,#], Adrian Frick[1,#], Bernhard Scheiner[1,2], Maximilian Baumgartner[3], Klaus G. Schmetterer[4], Carlos S. Reyna-Blanco[5], Lorenz Balcar[1,2], Larissa Pajancic[1,2], Abelina Kreuter[1,2], Joana Séneca[6,7,8], Bela Hausmann[7,8], Thomas Köcher[9], Melanie Prinzensteiner[5], Katharina Lampichler[10], Nataliya Rohr-Udilova[1,2], Adrija Chakrabarty[3], Anton Klotz[1], Annika C. Lampl[4], Kerstin Zinober[1], Katharina Regnat[1], Michael Trauner[1], David Berry[6,7], Dietmar Tamandl[10], Thomas Vogl[5], Christoph Gasche[1], Matthias Pinter[1,2,*]

[1] Division of Gastroenterology and Hepatology, Department of Medicine III, Medical University of Vienna, Vienna, Austria

[2] Vienna Liver Cancer Study Group, Division of Gastroenterology and Hepatology, Department of Medicine III, Medical University of Vienna, Vienna, Austria

[3] CeMM Research Center for Molecular Medicine of the Austrian Academy of Sciences

[4] Department of Laboratory Medicine, Medical University of Vienna, Vienna, Austria

[5] Center for Cancer Research, Medical University of Vienna, Vienna, Austria

[6] Centre for Microbiology and Environmental Systems Science, Department of Microbiology and Ecosystem Science, Division of Microbial Ecology, University of Vienna, Vienna, Austria.

[7] Joint Microbiome Facility of the Medical University of Vienna and the University of Vienna, Vienna, Austria.

[8] Division of Clinical Microbiology, Department of Laboratory Medicine, Medical University of Vienna, Vienna, Austria

[9] Metabolomics Service and Research Facility, Vienna Biocenter Core Facilities, Vienna, Austria

[10] Department of Biomedical Imaging and Image-guided Therapy, Medical University of Vienna, Vienna, Austria

[#] These authors contributed equally to this work.

*** Correspondence to:**

Matthias Pinter

Division of Gastroenterology and Hepatology

Department of Medicine III

Medical University of Vienna

Währinger Gürtel 18-20, 1090 Vienna, Austria

M: matthias.pinter@meduniwien.ac.at

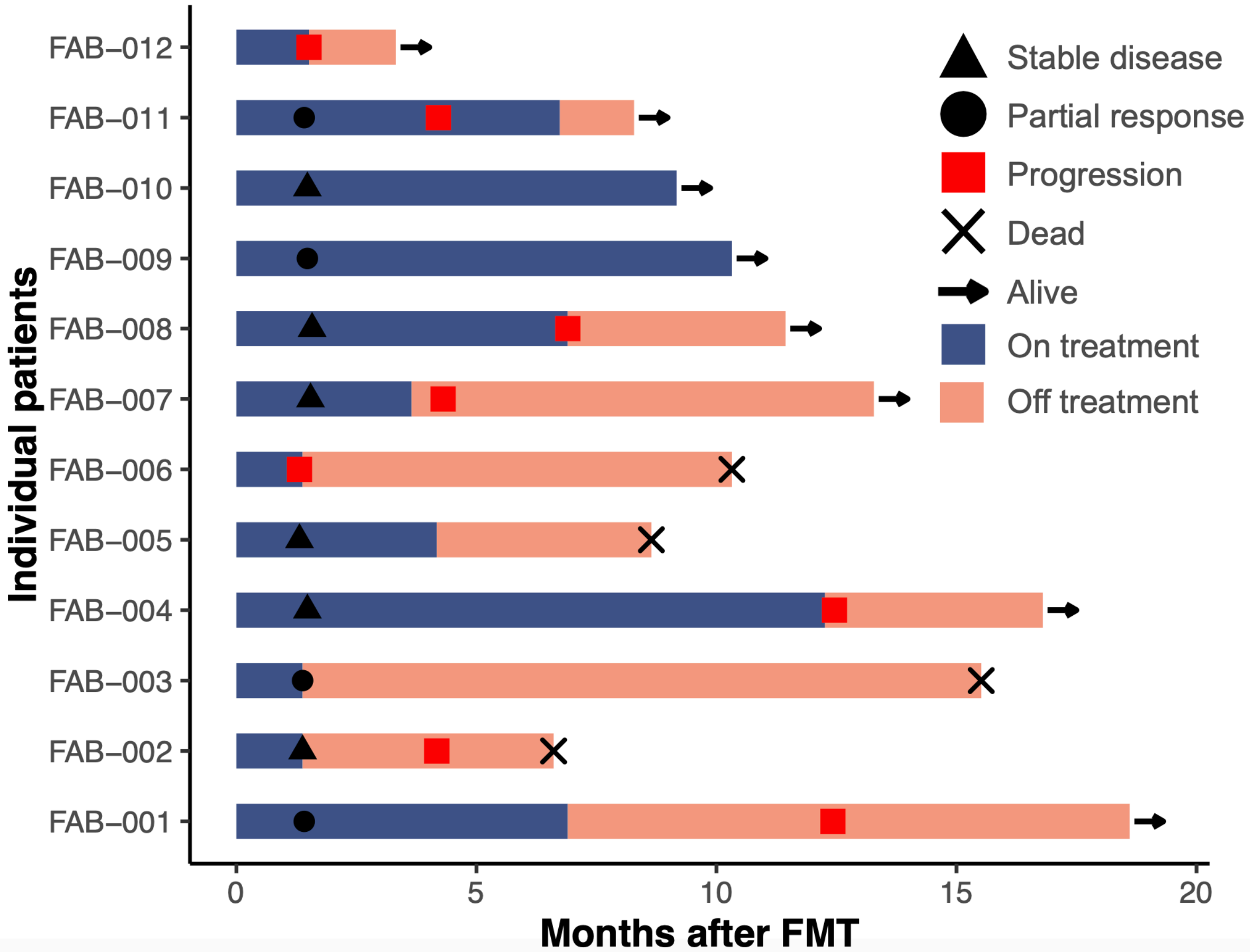

Supplementary Figure 1. Swimmer plot showing individual disease courses for all 12 patients included in the study. Radiological assessments were performed according to modified Response Evaluation Criteria in Solid Tumors.

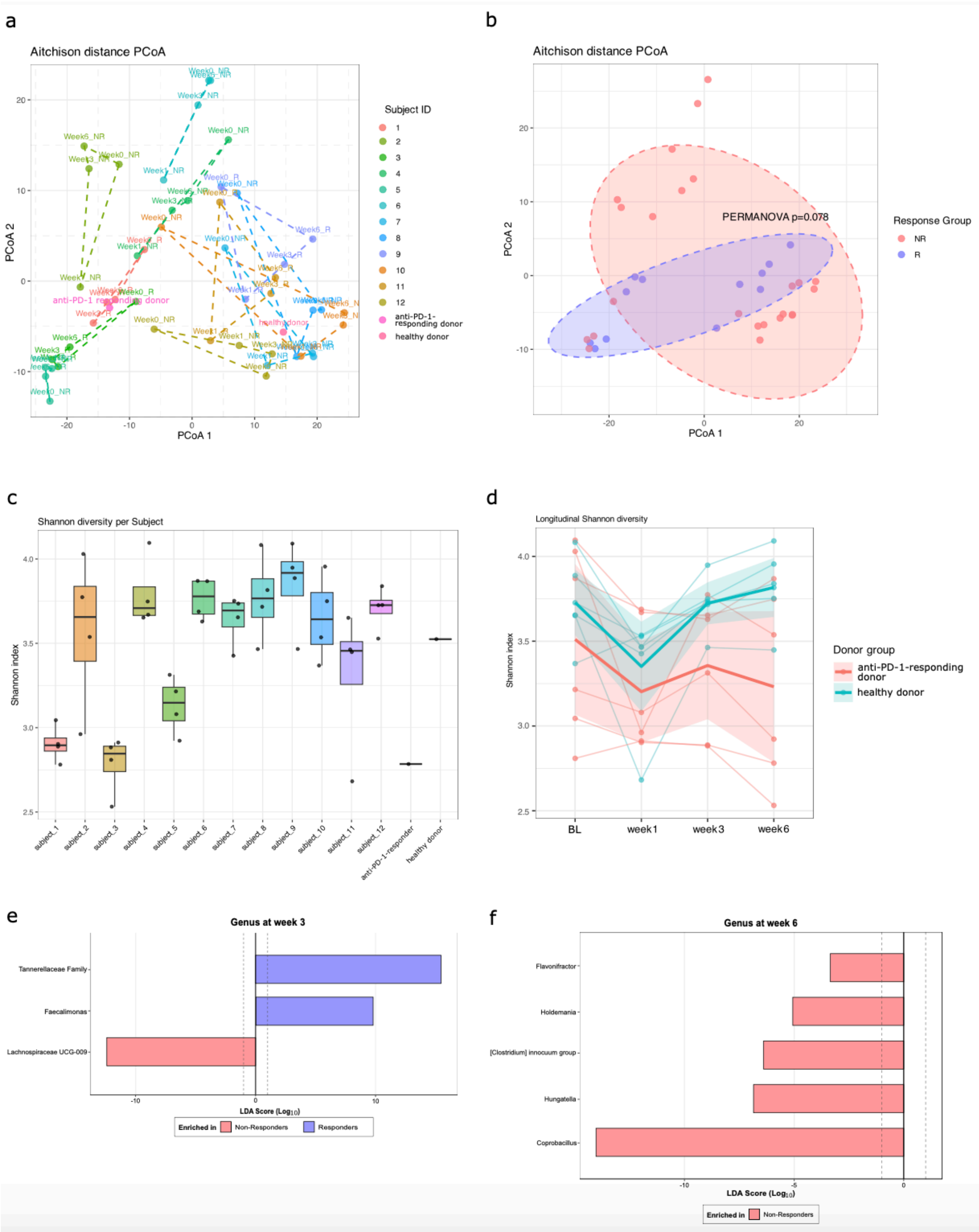

Supplementary Figure 2. Fecal microbial composition after fecal microbiota transplantation (FMT). a) Aitchison distance Principal Coordinates Analysis (PCoA) plot of bacterial composition (baseline (BL), weeks 1, 3, and 6) colored by subject (n=12) and donor (n=2). Donor samples represent the material used for FMT. NR, non-responder, R, responder. **b)** Aitchison distance PCoA plot of bacterial composition after FMT (weeks 1, 3, and 6) shows similar clustering by clinical response (PERMANOVA p=0.078), R (purple), n=4; NR (red), n=8). **c)** Shannon diversity by subject (BL, week 1, 3, and 6; each dot represents one time point/sample) and donor. Higher values indicate greater diversity. Box plots show the median (center line), 25th and 75th percentiles (box boundaries), and whiskers extending to the most extreme values within 1.5 *x* IQR; individual points represent individual

observations. **d)** Longitudinal Shannon diversity of patient microbiome in each individual patient, and according to donor group. Thick green and red lines represent mean Shannon diversity trajectories for donor groups (anti-PD-1-responding donor recipients (red), n=6; healthy donor recipients (turquois), n=6). The green and red shaded areas represent the standard deviation. **e, f)** LDA using linear discriminant analysis effect size (LEfSe) of differentially abundant taxa at the genus level between responders (n=4) and non-responders (n=8) at week 3 **(e)** and week 6 **(f)** after FMT; Kruskal Wallis, p value < 0.05 (two-sided).

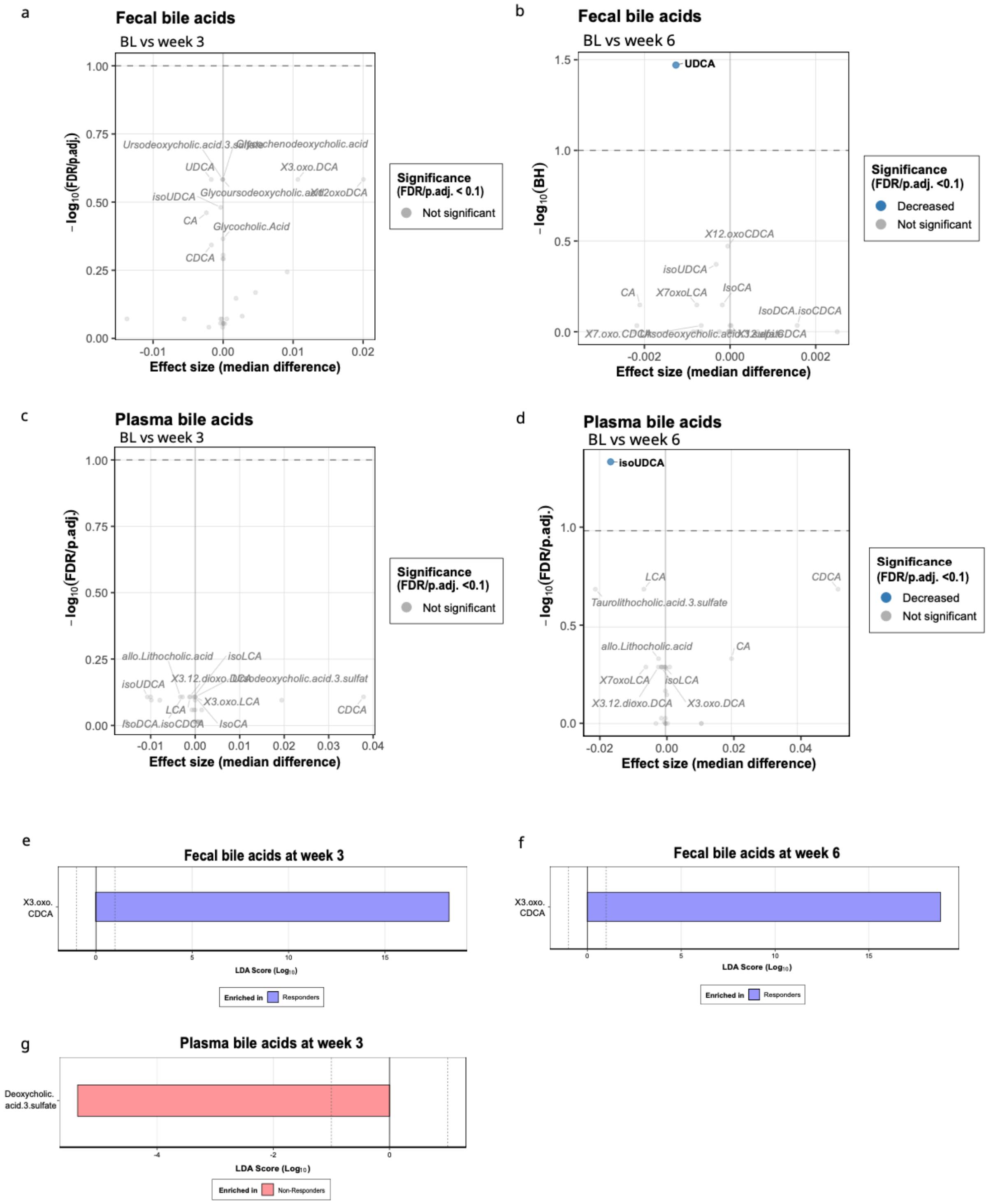

Supplementary Figure 3. Fecal and plasma bile acid metabolites profiling after fecal microbiota transplantation (FMT). a-d) Volcano plots illustrating changes of bile acid metabolites in stool at week 3 **(a)** and week 6 **(b)**, and in plasma at week 3 **(c)** and week 6 **(d)** after FMT compared to baseline (n=12). The x-axis represents median difference in total sum scaling (TSS)-normalized abundances, and the y-axis shows $-\log_{10}$ adjusted p-values (paired Wilcoxon signed rank test) after Benjamini-Hochberg (BH) correction, with higher values indicating greater statistical significance, significance threshold: False Discovery Rate (FDR) < 0.1 (two-sided). **e-g)** LDA using linear discriminant analysis effect size (LEfSe) of differentially abundant bile acid metabolites between responders (n=4) and non-responders (n=8) in stool at week 3 **(e)** and week 6 **(f),** and in plasma at week 3 **(g)** after FMT; Kruskal Wallis, p value < 0.05 (two-sided).

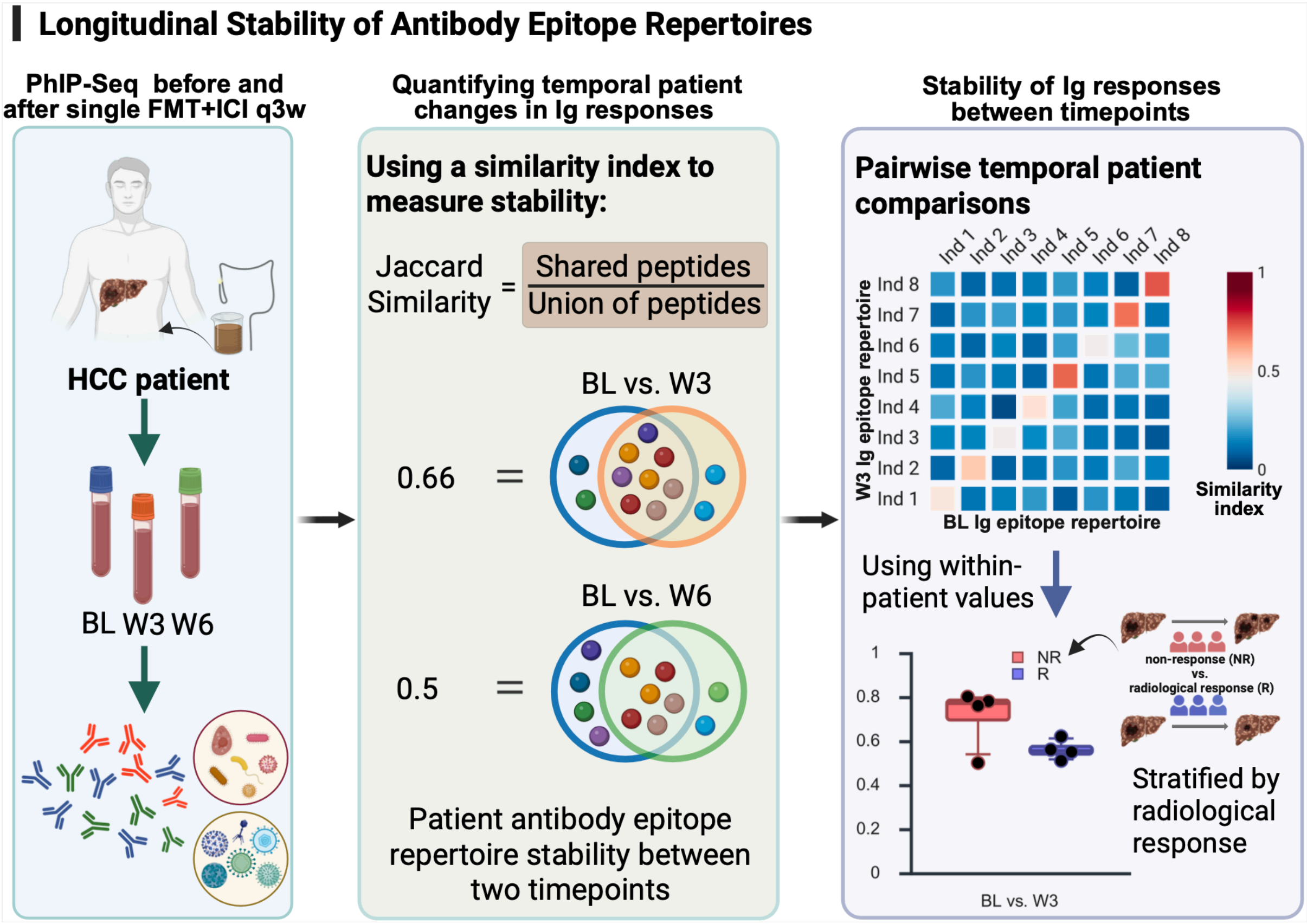

Supplementary Figure 4. Schematic overview of longitudinal stability analysis of antibody epitope repertoires profiled by phage immunoprecipitation sequencing (PhIP-Seq). Plasma samples were collected from patients with hepatocellular carcinoma (HCC) at baseline (BL) and at week 3 (W3) and week 6 (W6) after a single fecal microbiota transplantation (FMT) combined with the immune checkpoint inhibitor (ICI)-based combination of atezolizumab plus bevacizumab every 3 weeks (q3w). Antibody epitope repertoires were defined based on enriched peptides detected at each time point. Temporal stability was quantified using the Jaccard similarity index, calculated as the ratio of shared peptides to the union of peptides between two time points for the same individual (for example, BL vs. W3 or BL vs. W6). Pairwise similarity values were computed for all temporal comparisons, generating a similarity matrix across patients and time points. For downstream analyses, within-patient similarity values were extracted and summarized to compare repertoire stability between time points and to assess differences stratified by radiological response (responders, R; non-responders, NR).
Created with BioRender. Vogl, T. (2026) https://BioRender.com/sjiufpi.

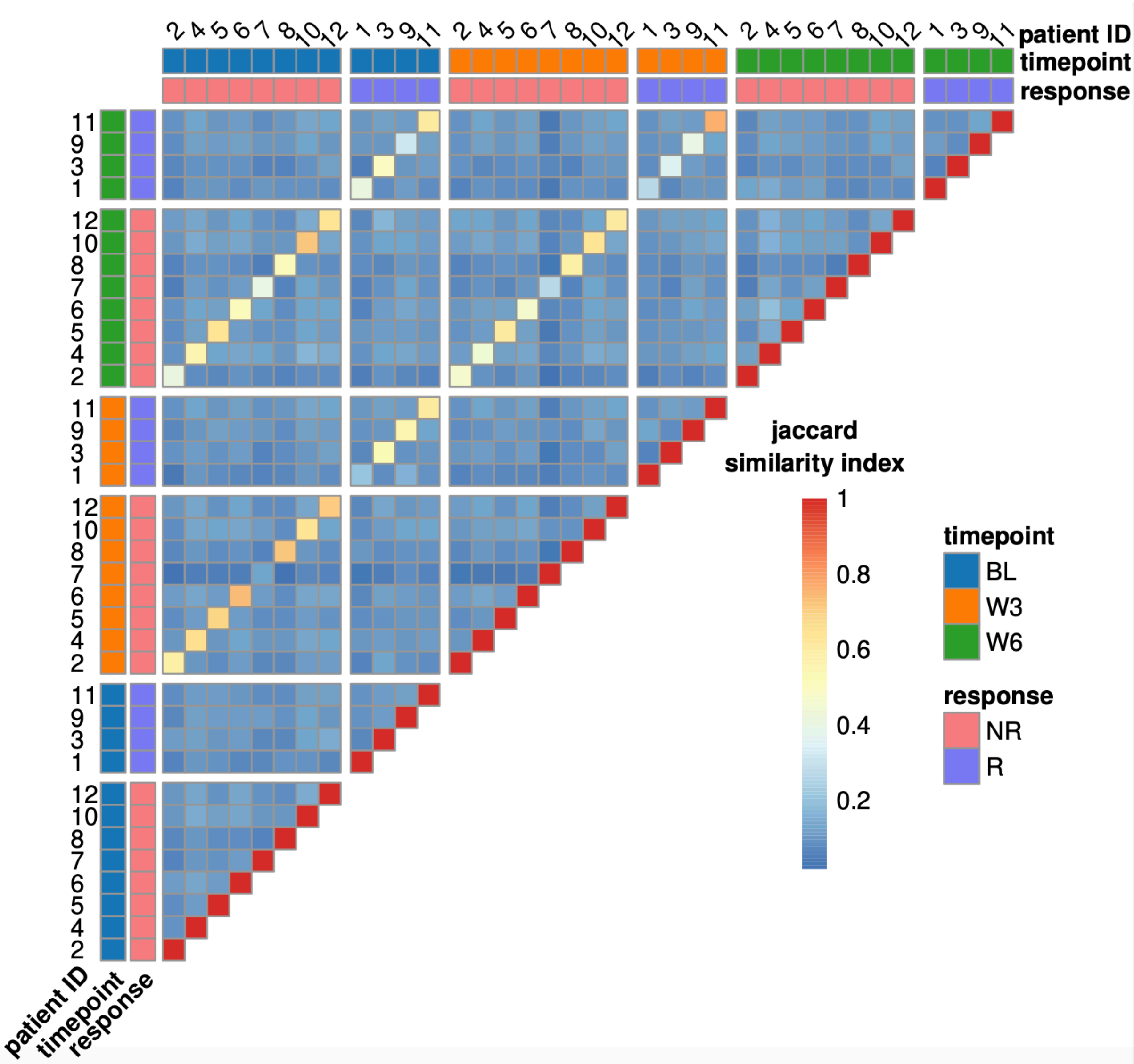

Supplementary Figure 5. Pairwise similarity of antibody epitope repertoires across patients, time points, and radiological response. Heatmap showing pairwise Jaccard similarity indices calculated between antibody epitope repertoires derived from PhIP-Seq across all patient samples. Each tile represents the similarity between two samples, with higher values (red) indicating greater overlap of enriched peptides and lower values (blue) indicating reduced similarity. Samples are ordered and grouped by time point (baseline [BL], week 3 [W3], week 6 [W6]) and radiological response (responders, R, n=4; non-responders, NR, n=8), as indicated by the annotation bars. The diagonal corresponds to self-comparisons (Jaccard similarity = 1) and is shown for reference, while only the upper triangular portion of the similarity matrix is displayed to avoid redundancy.

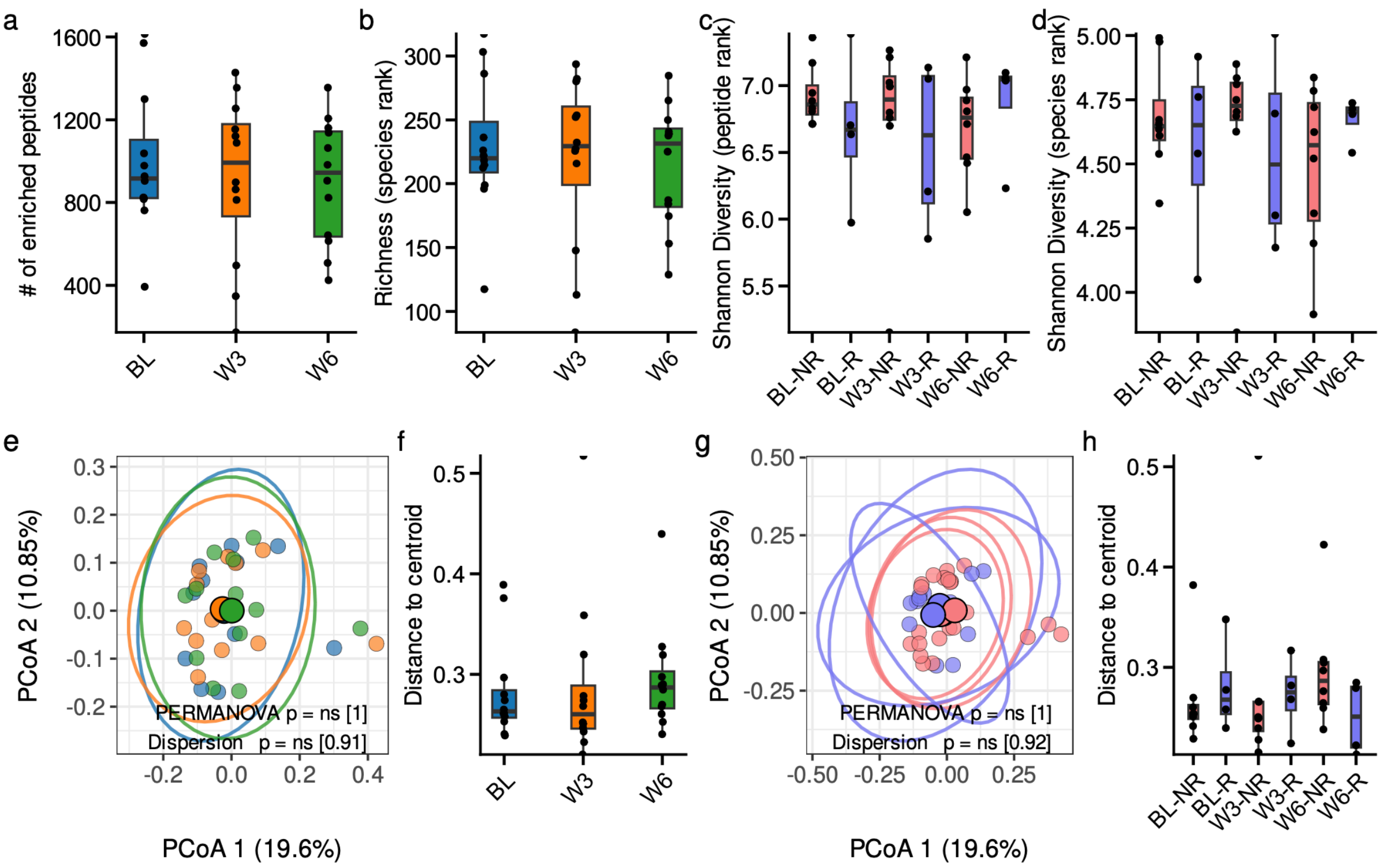

Supplementary Figure 6. Longitudinal composition and diversity of antibody epitope repertoires profiled by phage immunoprecipitation sequencing (PhIP-Seq). a) Number of enriched peptides detected by PhIP-Seq at baseline (BL), week 3 (W3), and week 6 (W6) (n=12). **b)** Antigenic richness at each time point, expressed as the rank of detected species (n=12). **c)** Shannon diversity index at the peptide level, stratified by radiological response (responders, R, n=4; non-responders, NR, n=8). **d)** Shannon diversity index at the species level, stratified by radiological response (R, n=4; NR, n=8) and time point. **e)** Principal coordinate analysis (PCoA) based on Bray-Curtis dissimilarity computed from relative species abundance matrices, illustrating overall similarity of antibody repertoire composition across time points (BL, W3, W6) (n=12). Ellipses denote 95% confidence intervals around group centroids. Permutational multivariate analysis of variance (PERMANOVA) and dispersion tests indicate no significant differences between time points. **f)** Distance of each sample to its group centroid in PCoA space, summarizing within-group dispersion across time points (n=12). **g)** PCoA based on Bray-Curtis dissimilarity of relative species abundances, stratified by radiological response (R, n=4; NR, n=8) at each time point, with 95% confidence ellipses shown. PERMANOVA and dispersion testing indicate no significant separation by response status. **h)** Distance to centroid for responder (n=4) and non-responder (n=8) groups at each time point, reflecting comparable within-group dispersion. Box plots in panels a-d, f, and h show the median (center line), 25th and 75th percentiles (box boundaries), and whiskers extending to the most extreme values within 1.5 *x* IQR; individual points represent individual observations.

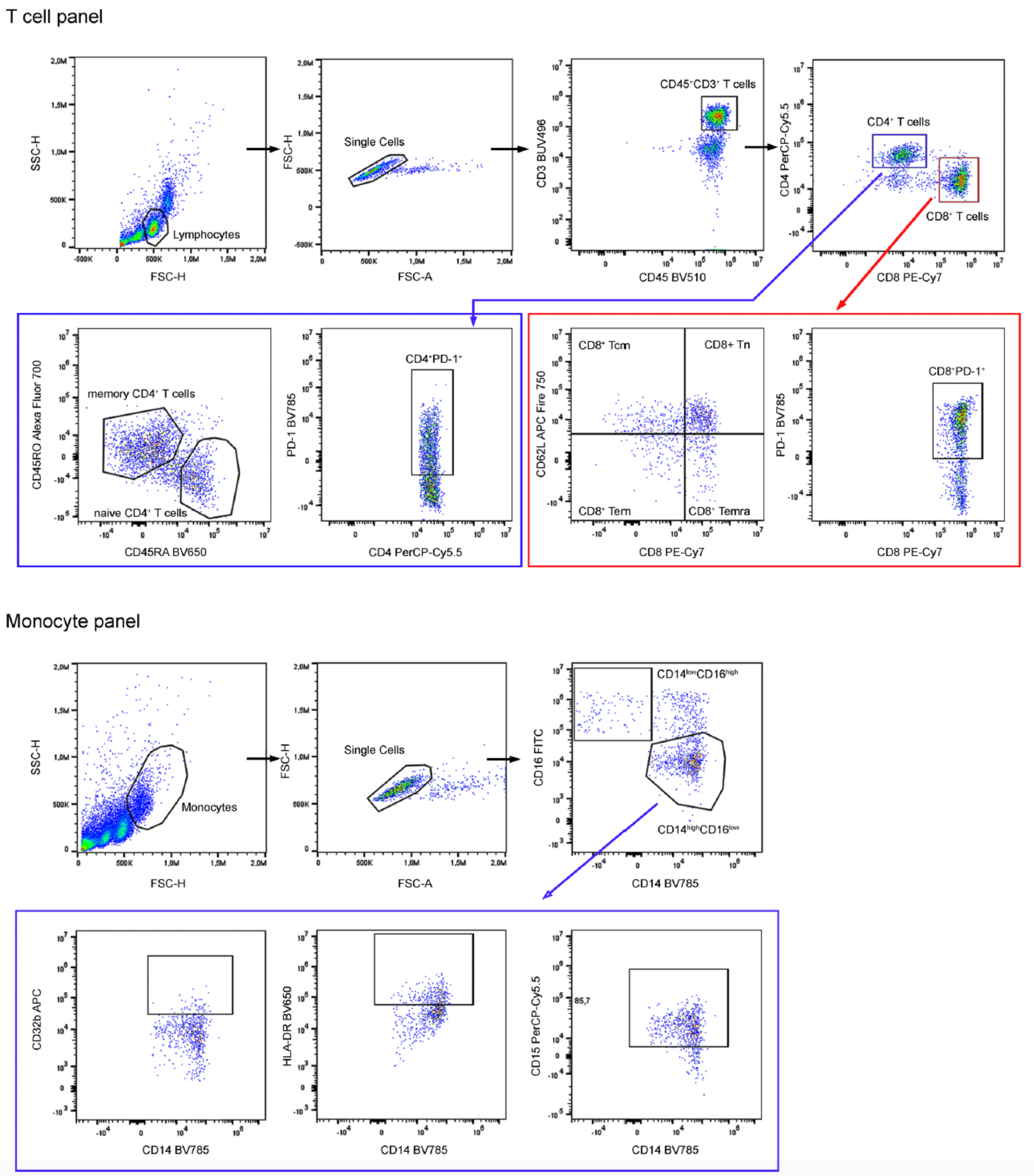

Supplementary Figure 7. Flow cytometry gating strategy for T cells and monocytes. The gating strategy for T cells corresponds to Figure 4 a (left panel), b, and c. The gating strategy for monocytes corresponds to Figure 4 a (right panel), d, and e.

Supplementary Table 1. Clinical characteristics of individual patients

Patient #	Donor group	BOR to prior AB	Time from last dose of prior A to trial FMT, months	Therapies between start of prior AB and study inclusion	BOR in current clinical trial of FMT combined with AB	
					mRECIST	RECIST v1.1
FAB-001	Anti-PD-1-R	PR	1.3	–	PR	PR
FAB-002	Anti-PD-1-R	SD	19.5	Lenvatinib, cabozantinib, sorafenib, regorafenib	SD	SD
FAB-003	Anti-PD-1-R	PR	13.9	–	PR	PR
FAB-004	Anti-PD-1-R	PD	0.8	–	SD	SD
FAB-005	Anti-PD-1-R	PD	0.9	–	SD	SD
FAB-006	Anti-PD-1-R	PD	0.7	–	PD	PD
FAB-007	Healthy	PD	18.2	Lenvatinib, sorafenib, ramucirumab, cabozantinib, RT of lung metastasis, regorafenib	SD	SD
FAB-008	Healthy	PR	0.7	Intrahepatic RT during AB	SD	SD
FAB-009	Healthy	PD	1.1	–	PR	SD
FAB-010	Healthy	SD	14.0	Lenvatinib, sorafenib	SD	SD
FAB-011	Healthy	PR	9.2	Sorafenib, adrenalectomy	PR	PR
FAB-012	Healthy	PD	1.2	–	PD	PD

Abbreviations: A, atezolizumab; AB, atezolizumab plus bevacizumab; Anti-PD-1-R, anti-programmed cell death protein 1 responder; BOR, best overall response; FMT, fecal microbiota transplantation; mRECIST, modified Response Evaluation Criteria in Solid Tumors; PD, progressive disease; PR, partial response; RECIST v1.1, Response Evaluation Criteria in Solid Tumors version 1.1.; RT, radiation therapy; SD, stable disease.

Supplementary Table 2. Clinical characteristics of FMT donors

Donor	Age (years)	Sex	Relevant medical conditions	HCC stage	Anti-PD-1 therapy	Time in PR
Anti-PD-1-R	>75	female	HCC, cirrhosis, history of cured HCV	BCLC C	Nivolumab	>5 years
Healthy	<30	male	–	–	–	–

Abbreviations: Anti-PD-1, anti-programmed cell death protein 1; Anti-PD-1-R, anti-programmed cell death protein 1 responder; BCLC, Barcelona Clinic Liver Cancer; FMT, fecal microbiota transplantation; HCC, hepatocellular carcinoma; HCV, hepatitis C virus; PR, partial response.

Supplementary Table 3. Protocol amendments

Amendment No.	Date	Section	Main change	Rationale	Impact
1	05/NOV/2022	Safety definitions and reporting	Information on AE, AESI, and pregnancy reporting was added	To clarify safety reporting	Improves consistency of safety reporting
2	23/MAY/2023	Inclusion criteria	Allow inclusion of patients with prior response	To improve recruitment rate	Broadens patient population
3	17/OCT/2023	Donor selection criteria	Recipient-donor sero-matching requirement was removed	Not recommended by international guidelines	Broadens patient population
4	02/NOV/2023	Donor selection criteria	Allow healthy donor FMT	To expand donor availability	Expands donor pool
5	01/DEC/2023	Study personnel	New study staff was added	Ensure adequate staffing	No impact on study outcomes
6	11/JUN/2024	Study personnel	New study staff was added	Ensure adequate staffing	No impact on study outcomes
7	01/OCT/2024	Regulatory	Additional study documentation was submitted to ethics committee	To ensure regulatory compliance	No impact on study outcomes

Abbreviations: AE, adverse event; AESI, adverse events of special interest; FMT, fecal microbiota transplantation.

Supplementary Table 4. List of antibodies used for flow cytometry staining of peripheral blood mononuclear cells (PBMCs)

Targeting	Fluorochrome	Manufacturer	Cat #	Clone	Lot
CD3	BUV496	BD	569786	SK7	5066084
CD4	PerCP	Biolegend	317431	OKT4	B464075
CD8	PE-Cy7	Biolegend	344711	SK1	B431460
CD45RA	BV650	Biolegend	304135	HI100	B429182
CD45RO	Alexa Fluor 700	Biolegend	304217	UCHL1	B418391
CD62L	APC Fire 750	Biolegend	304845	DREG-56	B380595
CD279 (PD-1)	BV785	Biolegend	329929	EH12.2H7	B419634
CD14	BV785	Biolegend	325643	HCD14	B430184
CD15	PerCP	Biolegend	323018	W6D3	B403065
CD16	FITC	Biolegend	302005	3G8	B412161
CD32b	APC	AntibodySystem	ANSY-FHD97923	6G08	N/A
CD45	BV510	Biolegend	304035	HI30	B426007
HLA-DR	BV650	Biolegend	307649	L243	B435851
