# Supplementary material for: FMT combined with atezolizumab-bevacizumab in hepatocellular carcinoma refractory to immunotherapy: an open label, single-arm, phase II pilot study
Source: Nat Commun. 2026 Oct 7;17:11442. doi: 10.1038/s41467-026-78219-z (PMC13736962; doi:10.1038/s41467-026-78219-z)
Supplement: Supplementary file 2 [file 41467_2026_78219_MOESM2_ESM.pdf]

nature portfolio

Corresponding author(s): Matthias Pinter

Last updated by author(s): Sep 3, 2026

Reporting Summary

Nature Portfolio wishes to improve the reproducibility of the work that we publish. This form provides structure for consistency and transparency in reporting. For further information on Nature Portfolio policies, see our Editorial Policies and the Editorial Policy Checklist.

Statistics

For all statistical analyses, confirm that the following items are present in the figure legend, table legend, main text, or Methods section.

n/a	Confirmed	
☐	☒	The exact sample size (*n*) for each experimental group/condition, given as a discrete number and unit of measurement
☐	☒	A statement on whether measurements were taken from distinct samples or whether the same sample was measured repeatedly
☐	☒	The statistical test(s) used AND whether they are one- or two-sided<br>*Only common tests should be described solely by name; describe more complex techniques in the Methods section.*
☒	☐	A description of all covariates tested
☐	☒	A description of any assumptions or corrections, such as tests of normality and adjustment for multiple comparisons
☐	☒	A full description of the statistical parameters including central tendency (e.g. means) or other basic estimates (e.g. regression coefficient) AND variation (e.g. standard deviation) or associated estimates of uncertainty (e.g. confidence intervals)
☐	☒	For null hypothesis testing, the test statistic (e.g. *F*, *t*, *r*) with confidence intervals, effect sizes, degrees of freedom and *P* value noted<br>*Give P values as exact values whenever suitable.*
☒	☐	For Bayesian analysis, information on the choice of priors and Markov chain Monte Carlo settings
☒	☐	For hierarchical and complex designs, identification of the appropriate level for tests and full reporting of outcomes
☐	☒	Estimates of effect sizes (e.g. Cohen's *d*, Pearson's *r*), indicating how they were calculated

Our web collection on statistics for biologists contains articles on many of the points above.

Software and code

Policy information about availability of computer code

Data collection

Clinical data were collected and entered in the electronic data capturing system 'clincase' (Version 2.7.0.12, Quadratek Data Solutions Ltd.). DNA extraction, 16S rRNA gene sequencing, and raw data processing was performed at the Joint Microbiome Facility of the Medical University of Vienna and the University of Vienna (project ID JMF-2401-16) . Amplicon pools were extracted from the raw sequencing data using the FASTQ workflow in BaseSpace (Illumina) with default parameters. Demultiplexing was performed with the python package demultiplex (Laros JFJ, github.com/jfjlaros/demultiplex) allowing one mismatch for barcodes and two mismatches for linkers and primers. Amplicon sequence variants (ASVs) were inferred using the DADA2 R package v1.35.8. ASV sequences were subsequently classified using DADA2 and the SILVA database SSU Ref NR 99 release 138.2.
Bile acids and their conjugates were measured with reversed phase Liquid Chromatography-Tandem Mass Spectrometry (LC-MS/MS). Retention times, SRM-transitions, and optimal collisional energies were determined by authentic standards. All data interpretation was performed using Xcalibur (Thermo Fisher Scientific).
PBMCs were analysed on a CytoFlex LX flow cytometer (Beckman Coulter, Brea, CA, USA) at the Core Facility Flow Cytometry of the Medical University of Vienna. Subsequently, T cell and monocyte phenotypes were analyzed using FlowJo_v10.10.0 software (Beckton Dickinson, Franklin Lakes, NJ, USA).
PhIP Seq: Three antigen libraries covering a total of 357,000 peptide sequences were used for phage immunoprecipitation sequencing. Single-end 75-nt Illumina sequencing was performed for all plasma samples as well as for the input library, defined as sequencing of the antigen library prior to immunoprecipitation. Raw sequencing reads spanning the peptide-identifying barcode (75 nt) were aligned to a reference database of barcode sequences corresponding to expressed peptides using Bowtie2, allowing up to two mismatches. Reads mapping to each barcode were quantified using SAMtools and awk. Enriched clones were identified by fitting a three-parameter zero-inflated generalized Poisson (ZIGP) distribution to read counts from clones sharing identical input library counts. Prior to parameter estimation, the top 1% of read counts within each input count bin were excluded to reduce the influence of extreme values. ZIGP parameters were modeled as functions of input read count by fitting cubic splines constrained to be strictly increasing for parameters λ and θ, and strictly decreasing for parameter π,

using the scam package in R. Peptides were considered significantly enriched if their observed read counts corresponded to a p value ≤ 0.05 after Holm correction for multiple testing. For downstream analyses, antibody epitope repertoires were represented as binary matrices indicating the presence or absence of enriched peptides for each sample, as defined by the enrichment procedure described above.

Data analysis

Statistical analyses of clinical data were performed using IBM SPSS Statistics 30 (SPSS Inc., Armonk, New York, USA), R 4.3.1 (R Core Team, R Foundation for Statistical Computing, Vienna, Austria) and GraphPad Prism 9 (GraphPad Software, La Jolla, California, USA).
For fecal microbiota analysis, downstream analyses were performed with R version 4.4.2 using vegan (v2.6-8), phyloseq (v1.50.0), and DESeq2 (v1.46.0). Aitchison distances were calculated from (clr+1) transformed ASV counts and analyzed by PERMANOVA (adonis2). Bray-Curtis distances between donor and recipient were calculated using phyloseq and total sum scaled ASV tables. Differential abundance was assessed at genus level using DESeq2 with paired design (~ subject + timepoint), local dispersion fitting, and Wald test. Statistical significance was defined by a false discovery rate (FDR) of < 0.1, adjusted by Benjamini-Hochberg correction; effect sizes are log2 fold-changes. Linear discriminant analysis effect size (LEfSe) was performed to identify bacterial genera differently abundant between response groups at week 3 and 6, using Kruskal-Wallis test (α = 0.05, two-sided) for statistical significance.
Bile acid analysis (stool and plasma) was performed in R version 4.4.2. Metabolites were filtered (prevalence ≥5%, variance >25th percentile) and normalized by total sum scaling. Differences between baseline and post-FMT timepoints (week 3, week 6) were assessed using paired Wilcoxon signed rank tests. Statistical significance was defined by a FDR of < 0.1 (two-sided), adjusted by Benjamini-Hochberg correction; effect sizes are median differences in normalized abundances. Linear discriminant analysis effect size (LEfSe) was performed to identify metabolites differently abundant between response groups at week 3 and 6, using Kruskal-Wallis test (α = 0.05, two-sided) for statistical significance.
PhIP Seq:To assess global similarity of antibody repertoires across samples, Bray-Curtis dissimilarities were computed from relative species abundance matrices using the vegdist function of the vegan R package (v.2.7.1). Principal coordinate analysis (PCoA) was performed on the resulting distance matrices. Group-level differences in repertoire composition were tested using permutational multivariate analysis of variance (PERMANOVA) implemented with the adonis2 function (1,000 permutations). Homogeneity of multivariate dispersion was assessed using the betadisper function followed by permutation testing (permutest, 1,000 permutations). Distances to group centroids in PCoA space were extracted to quantify within-group dispersion. Longitudinal stability of antibody epitope repertoires was quantified using the Jaccard similarity index using the binary peptide enrichment data. Pairwise Jaccard distances were computed using vegdist (method = "jaccard"), and transformed to similarity values as 1 − distance. Within-patient similarity values were extracted by comparing samples collected from the same individual at different time points (baseline vs. week 3 and baseline vs. week 6). These within-individual similarity values were summarized using boxplots and, where indicated, stratified by radiological response. Differences in Jaccard similarity indices were assessed using aligned rank transform (ART) models implemented in the ARTool R package, allowing non-parametric testing of factorial effects involving time point and radiological response. Post hoc contrasts were computed using art.con. For comparisons stratified by response, p values were adjusted using the Benjamini-Hochberg procedure. All analyses were performed in R (v4.5.1) on Ubuntu 24.04.3 LTS. All data visualizations were generated using ggplot2 (v.3.5.2).

For manuscripts utilizing custom algorithms or software that are central to the research but not yet described in published literature, software must be made available to editors and reviewers. We strongly encourage code deposition in a community repository (e.g. GitHub). See the Nature Portfolio guidelines for submitting code & software for further information.

Data

Policy information about availability of data

All manuscripts must include a data availability statement. This statement should provide the following information, where applicable:

- Accession codes, unique identifiers, or web links for publicly available datasets
- A description of any restrictions on data availability
- For clinical datasets or third party data, please ensure that the statement adheres to our policy

The data generated and analyzed during this study are available within the article, its supplementary information, and publicly accessible repositories. The minimum dataset used for the primary, secondary, and exploratory analyses may be shared with qualified medical or scientific professionals for a defined research purpose. Deidentified individual participant data may be provided after a data sharing agreement has been signed. All requests for data access should be sent to the corresponding author (MP). Requests will be considered and responded to within 2 months of receipt. Patient-related data not included in the paper were generated as part of a clinical trial and are subject to patient confidentiality.
The 16S rRNA gene amplicon sequencing data has been deposited at the National Center for Biotechnology Information (NCBI) Sequence Read Archive, BioProject ID PRJNA1444467. The PhIP-Seq data and associated metadata used in this study are publicly available in the FAB-HCC repository at https://github.com/csReynaB/FAB-HCC. A permanent archived version of the repository associated with this study is available through Zenodo at https://doi.org/10.5281/zenodo.21921459. The targeted metabolomics data for this study have been deposited to MetaboLights repository of the European Bioinformatics Institute (EMBL-EBI) with the dataset identifier MTBLS12023, available at https://www.ebi.ac.uk/metabolights/MTBLS12023.

Research involving human participants, their data, or biological material

Policy information about studies with human participants or human data. See also policy information about sex, gender (identity/presentation), and sexual orientation and race, ethnicity and racism.

Reporting on sex and gender

Biological sex was recorded for all participants at enrollment. Gender identity was not recorded. Sex was not used as an inclusion or exclusion criterion. Since the study was not powered to evaluate sex-based differences, no analyses stratified by sex were performed.

Reporting on race, ethnicity, or other socially relevant groupings

We did not report on race, ethnicity, or any socially relevant groupings in our study.

Population characteristics

We enrolled patients with advanced hepatocellular carcinoma. Detailed population characteristics are provided in Table 1.

Recruitment	Patients were enrolled and treated at the Division of Gastroenterology & Hepatology, Medical University of Vienna. Patients were either screened and recruited at our outpatient clinic or referred from other hospitals in Vienna and the surrounding areas.
Ethics oversight	The study was approved by the Ethics Committee of the Medical University of Vienna (1054/2022) in accordance with applicable Austrian regulations and the requirements of the Austrian Federal Office for Safety in Health Care (BASG). The conduct of the study complied with the Declaration of Helsinki and the principles of Good Clinical Practice.

Note that full information on the approval of the study protocol must also be provided in the manuscript.

Field-specific reporting

Please select the one below that is the best fit for your research. If you are not sure, read the appropriate sections before making your selection.

☒ Life sciences ☐ Behavioural & social sciences ☐ Ecological, evolutionary & environmental sciences

For a reference copy of the document with all sections, see nature.com/documents/nr-reporting-summary-flat.pdf

Life sciences study design

All studies must disclose on these points even when the disclosure is negative.

Sample size	As this study was designed as a pilot study evaluating safety and feasibility, and because of its single-arm design, no formal sample size calculation was performed. The predefined sample size of this trial was 12 patients. The rationale was that at the time this study was designed, no comparable studies in HCC had been published, and the available evidence was limited to two studies evaluating safety and efficacy of FMT in immunotherapy-refractory patients with advanced melanoma, which included a comparable sample size (n=10 and 16). Thus, the number of patients was expected to sufficiently fulfill the pilot study's main objectives. No inferential statistical testing was used for calculation of primary safety and secondary efficacy endpoints.
Data exclusions	All 12 patients were included in safety and efficacy analyses.
Replication	This is a phase II pilot trial with a sample size of 12 patients, thus, the results need to be confirmed in a larger (ideally randomized) trial.
Randomization	This was a single-arm study, no randomization was done.
Blinding	This was an open-label, single arm trial, no blinding was performed.

Reporting for specific materials, systems and methods

We require information from authors about some types of materials, experimental systems and methods used in many studies. Here, indicate whether each material, system or method listed is relevant to your study. If you are not sure if a list item applies to your research, read the appropriate section before selecting a response.

Materials & experimental systems

n/a	Involved in the study	
☐	☒	Antibodies
☒	☐	Eukaryotic cell lines
☒	☐	Palaeontology and archaeology
☒	☐	Animals and other organisms
☐	☒	Clinical data
☒	☐	Dual use research of concern
☒	☐	Plants

Methods

n/a	Involved in the study	
☒	☐	ChIP-seq
☐	☒	Flow cytometry
☒	☐	MRI-based neuroimaging

Antibodies

Antibodies used	Antibodies for flow cytometry are provided in Supplementary Table 4.
Validation	Validation is provided on the manufacturer's website:<br>https://www.bdbiosciences.com/en-eu/products/reagents/flow-cytometry-reagents/research-reagents/single-color-antibodies-ruo/buv496-mouse-anti-human-cd3.569786?tab=product_details<br>https://www.biolegend.com/de-de/clone-search/percp-anti-human-cd4-antibody-6376<br>https://www.biolegend.com/fr-ch/products/pe-cyanine7-anti-human-cd8-antibody-6390<br>https://www.biolegend.com/en-gb/products/brilliant-violet-650-anti-human-cd45ra-antibody-7662<br>https://www.biolegend.com/fr-ch/products/alexa-fluor-700-anti-human-cd45ro-antibody-3422<br>https://www.biolegend.com/fr-lu/products/apc-fire-750-anti-human-cd62l-antibody-13524<br>https://www.biolegend.com/nl-nl/products/brilliant-violet-785-anti-human-cd279-pd-1-antibody-7980

https://www.biolegend.com/en-gb/products/brilliant-violet-785-anti-human-cd14-antibody-25020
https://www.biolegend.com/nl-be/products/percp-anti-human-cd15-ssea-1-antibody-4248
https://www.biolegend.com/fr-lu/products/fitc-anti-human-cd16-antibody-567
https://www.antibodysystem.com/product/25201.html
https://www.biolegend.com/ja-jp/products/brilliant-violet-510-anti-human-cd45-antibody-8006?GroupID=BLG5926
https://www.biolegend.com/fr-lu/explore-new-products/brilliant-violet-650-anti-human-hla-dr-antibody-8875

Clinical data

Policy information about clinical studies
All manuscripts should comply with the ICMJE guidelines for publication of clinical research and a completed CONSORT checklist must be included with all submissions.

Clinical trial registration: ClinicalTrials.gov identifier: NCT05750030

Study protocol: The study protocol is included in the submission

Data collection: At each clinical visit, patient data obtained from a complete clinical assessment and laboratory work-up were collected and entered in the electronic data capturing system 'clincase' (Version 2.7.0.12, Quadratek Data Solutions Ltd.).

Outcomes: The primary objective of this study was safety as measured by incidence and severity of treatment-related adverse events. Secondary objectives included assessment of best radiological response according to mRECIST and RECISTv1.1, objective response rate, disease-control rate, progression-free survival, and overall survival.

Plants

Seed stocks: *Report on the source of all seed stocks or other plant material used. If applicable, state the seed stock centre and catalogue number. If plant specimens were collected from the field, describe the collection location, date and sampling procedures.*

Novel plant genotypes: *Describe the methods by which all novel plant genotypes were produced. This includes those generated by transgenic approaches, gene editing, chemical/radiation-based mutagenesis and hybridization. For transgenic lines, describe the transformation method, the number of independent lines analyzed and the generation upon which experiments were performed. For gene-edited lines, describe the editor used, the endogenous sequence targeted for editing, the targeting guide RNA sequence (if applicable) and how the editor was applied.*

Authentication: *Describe any authentication procedures for each seed stock used or novel genotype generated. Describe any experiments used to assess the effect of a mutation and, where applicable, how potential secondary effects (e.g. second site T-DNA insertions, mosiacism, off-target gene editing) were examined.*

Flow Cytometry

Plots

Confirm that:

☒ The axis labels state the marker and fluorochrome used (e.g. CD4-FITC).

☒ The axis scales are clearly visible. Include numbers along axes only for bottom left plot of group (a 'group' is an analysis of identical markers).

☒ All plots are contour plots with outliers or pseudocolor plots.

☒ A numerical value for number of cells or percentage (with statistics) is provided.

Methodology

Sample preparation: Aliquots of cryopreserved PBMCs (1x107 cells/mL) were thawed in RPMI 1640 supplemented with 10% FBS, washed twice, and resuspended in a buffer (PBS containing 0.5% FBS and 0.05% NaN3; FACS buffer) (Merck Millipore, Burlington, MA, USA). 1 x 106 cells were incubated with Human TruStain FcX™ Fc Receptor Blocking Solution (Biolegend, San Diego, CA, USA) for 10 min and then stained with a multicolor antibody cocktail covering T cell markers or monocyte markers (Supplementary Table 4) for 30 min at 4°C. Then, cells were washed twice in FACS buffer and transferred to a 96-well plate for analysis on a CytoFlex LX flow cytometer (Beckman Coulter, Brea, CA, USA) using CytExpert Software V2.5 (Beckman Coulter) for acquisition at the Core Facility Flow Cytometry of the Medical University of Vienna.

Instrument: CytoFlex LX 21-color flow cytometer

Software: CytExpert Software V2.5 for acquisition and FlowJo V10.10.0 for analysis

Cell population abundance: A minimum of 50 cells that express a marker of interest were targeted for each subpopulation of cells during acquisition

Gating strategy: The gating strategy is provided in Supplementary Figure 7.

☒ Tick this box to confirm that a figure exemplifying the gating strategy is provided in the Supplementary Information.
