# Supplementary material for: FMT combined with atezolizumab-bevacizumab in hepatocellular carcinoma refractory to immunotherapy: an open label, single-arm, phase II pilot study
Source: Nat Commun. 2026 Oct 7;17:11442. doi: 10.1038/s41467-026-78219-z (PMC13736962; doi:10.1038/s41467-026-78219-z)
Supplement: Supplementary file 3 [file 41467_2026_78219_MOESM3_ESM.pdf]

nature portfolio

Peer Review File

FMT combined with atezolizumab-bevacizumab in hepatocellular carcinoma refractory to immunotherapy: an open label, single-arm, phase II pilot study

Corresponding Author: Professor Matthias Pinter

This manuscript has been previously reviewed at another journal. This document only contains information relating to versions considered at Nature Communications.

Version 0:

Reviewer comments:

Reviewer #2

(Remarks to the Author)
The authors have addressed some of my comments on their earlier version. I am still concerned about the study design and the potential implications. I suggest that the authors tone down their claims throughout the paper.

1. In the Abstract of the paper, after they mention that this is a single arm phase-II pilot study, the authors should note something to the effect that this is an exploratory hypothesis generating study based on small sample sizes and that includes confounders and hence findings of the study need to be taken with caution.

2. Small sample sizes, together confounders not only impact type II error (power) but they can also impact the type I error (false discoveries), especially in the context of microbiome studies. Statistical methods for microbiome data, such as PERMANOVA, Deseq2, zero inflated generalized Poisson model etc. are dependent on large sample sizes. Hence, I am not sure if I agree with the authors' claim on page 6: "Recipient microbiome shifted towards donor microbiome, and healthy-donor FMT was associated with higher engraftment. FMT led to enrichment of response-associated and loss of deleterious gut bacteria." Although on page 18 they do acknowledge limitations of this study, the authors should tone down the above statements.

Reviewer #3

(Remarks to the Author)
General comments
The authors provided additional information and acknowledged several limitations. However, the major concerns remain only partially addressed, as outlined below.

Specific comments
1. It is still unclear whether the clinical and immunologic effects are attributable to FMT given the single-arm, non-randomized, uncontrolled design with a very small sample size.
2. The definition of refractoriness to atezolizumab plus bevacizumab is elusive. The intervals from the last dose to FMT vary from 0.7 to 19.5 months, during which several patients received multiple systemic and/or local therapies that act as significant confounding factors and obscure the true response.
3. The microbiome and immune analyses remain descriptive and underpowered, and won't establish mechanistic links between FMT, immune modulation, and clinical response.

Reviewer #4

(Remarks to the Author)
My previous concerns have been addressed and I don't have additional comment.

Open Access This Peer Review File is licensed under a Creative Commons Attribution 4.0 International License, which permits use, sharing, adaptation, distribution and reproduction in any medium or format, as long as you give appropriate credit to the original author(s) and the source, provide a link to the Creative Commons license, and indicate if changes were made.
In cases where reviewers are anonymous, credit should be given to 'Anonymous Referee' and the source.
The images or other third party material in this Peer Review File are included in the article’s Creative Commons license, unless indicated otherwise in a credit line to the material. If material is not included in the article’s Creative Commons license and your intended use is not permitted by statutory regulation or exceeds the permitted use, you will need to obtain permission directly from the copyright holder.
To view a copy of this license, visit https://creativecommons.org/licenses/by/4.0/

REVIEWERS' COMMENTS

Reviewer #2 (Remarks to the Author):

The authors have addressed some of my comments on their earlier version. I am still concerned about the study design and the potential implications. I suggest that the authors tone down their claims throughout the paper.

R: We thank the respected reviewer again for carefully evaluating our manuscript. As suggested, we have toned down any claims linking FMT with causality.

1. In the Abstract of the paper, after they mention that this is a single arm phase-II pilot study, the authors should note something to the effect that this is an exploratory hypothesis generating study based on small sample sizes and that includes confounders and hence findings of the study need to be taken with caution.

R: We have included in the Abstract that this was a hypothesis-generating pilot study, and acknowledged the small sample size as a limitation:

"In this single-center, single-arm phase II hypothesis-generating pilot study,…"

"Given the limited sample size, these findings need cautious interpretation and confirmation in larger, adequately powered trials."

2. Small sample sizes, together confounders not only impact type II error (power) but they can also impact the type I error (false discoveries), especially in the context of microbiome studies. Statistical methods for microbiome data, such as PERMANOVA, Deseq2, zero inflated generalized Poisson model etc. are dependent on large sample

sizes. Hence, I am not sure if I agree with the authors' claim on page 6: "Recipient microbiome shifted towards donor microbiome, and healthy-donor FMT was associated with higher engraftment. FMT led to enrichment of response-associated and loss of deleterious gut bacteria." Although on page 18 they do acknowledge limitations of this study, the authors should tone down the above statements.

R: As suggested, we have toned down these statements:

"Following FMT, recipient microbiome composition appeared more similar to that of the donor, and healthy donor FMT seemed to be associated with higher engraftment. FMT appeared to be associated with enrichment of response-associated and loss of deleterious gut bacteria."

Reviewer #3 (Remarks to the Author):

General comments

The authors provided additional information and acknowledged several limitations. However, the major concerns remain only partially addressed, as outlined below.

R: We thank the respected reviewer again for thouroughly evaluating our manuscript, and for their valuable input. We have addressed the remaining comments below.

Specific comments

1. It is still unclear whether the clinical and immunologic effects are attributable to FMT given the single-arm, non-randomized, uncontrolled design with a very small sample size.

R: All patients received the same immunotherapy regimen (i.e., atezolizumab plus bevacizumab) on which they had previously progressed. Thus, only FMT was the novel aspect of their treatment regimen. Given that the observed ORR was higher than what can be expected with continuation of immunotherapy beyond progression (Topp et al. Cancer Cell 2023; PMID: 37699333. Toh et al. J Clin Oncol 2022; DOI: 10.1200/JCO.2022.40.4_suppl.470), it seems reasonable to assume that FMT may have contributed to these findings. This is also in line with previous preclinical and clinical reports linking FMT to immunotherapy response (e.g., Gopalakrishnan et al. Science 2018; PMID: 29097493. Routy et al. Science; PMID: 29097494. Baruch et al. Science 2021; PMID: 33303685. Davar et al. Science 2021; PMID: 33542131). However, we agree with the reviewer that definitive proof requires a larger randomized controlled trial. We have acknowledged the hypothesis-generating nature of the study in the abstract and in the Limitations section of the Discussion:

Abstract:

"In this single-center, single-arm phase II hypothesis-generating pilot study,…"

"Given the limited sample size, these findings need cautious interpretation and confirmation in larger, adequately powered trials."

Discussion:

" … Given the limited sample size and the lack of a control group, the clinical findings of this study remain preliminary and warrant cautious interpretation. … The results from exploratory analyses (i.e., fecal microbiome analyses, bile acid profiling, immunophenotyping of immune cells, PhIP sequencing) are primarily descriptive and should therefore be considered hypothesis-generating."

2. The definition of refractoriness to atezolizumab plus bevacizumab is elusive. The intervals from the last dose to FMT vary from 0.7 to 19.5 months, during which several patients received multiple systemic and/or local therapies that act as significant confounding factors and obscure the true response.

R: Refractoriness was defined as progressive disease during prior treatment with atezolizumab plus bevacizumab. This definition has been provided in the Methods section under the Inclusion criteria: "Patients must have progressed (according to mRECIST) on previous treatment with atezolizumab/bevacizumab …". Patients who received other treatments after progressing on atezolizumab plus bevacizumab and before study inclusion (n=5) were allowed to participate. However, among the 4 responders in our trial, 3 did NOT receive any other treatment before study inclusion (see Supplementary Table 1).

We have added a statement to the Limitations section of the Discussion:

"Given the limited sample size and the lack of a control group, the clinical findings of this study remain preliminary and warrant cautious interpretation. The inclusion of patients who received other treatments after progressing on atezolizumab plus bevacizumab and before study inclusion may have introduced a potential confounding factor. …"

3. The microbiome and immune analyses remain descriptive and underpowered, and won't establish mechanistic links between FMT, immune modulation, and clinical response.

R: As stated above, this has been acknowledged in the Limitations section of the Discussion: "The results from exploratory analyses (i.e., fecal microbiome

analyses, bile acid profiling, immunophenotyping of immune cells, PhIP sequencing) are primarily descriptive and should therefore be considered hypothesis-generating."

Reviewer #4 (Remarks to the Author):

My previous concerns have been addressed and I don't have additional comment.

R: We sincerely thank the respected reviewer once again for their careful evaluation of our manuscript and for their constructive suggestions.
